# Exploratory Associations Between Climatic, Environmental, and Surveillance Indicators and Human West Nile Virus Infections in Apulia: A Bayesian Spatio-Temporal Analysis

**DOI:** 10.3390/v18090943

**Published:** 2026-08-28

**Authors:** Letizia Lorusso, Niccolò Maldera, Nicola Bartolomeo, Francesca Centrone, Maria Chironna, Paolo Trerotoli

**Affiliations:** 1 Department of Precision and Regenerative Medicine—(DiMePRe-J), University of Bari Aldo Moro, Piazza Giulio Cesare 11, 70124 Bari, Italy; 2 Department of Mathematics, University of Bari Aldo Moro, Piazza Giulio Cesare 11, 70124 Bari, Italy; n.maldera4@phd.uniba.it; 3 Department of Interdisciplinary Medicine, University of Bari Aldo Moro, Piazza Giulio Cesare 11, 70124 Bari, Italy; nicola.bartolomeo@uniba.it (N.B.); maria.chironna@uniba.it (M.C.); paolo.trerotoli@uniba.it (P.T.); 4 Laboratory of Public Health, AOUC Policlinico di Bari, 70124 Bari, Italy; francesca.centrone@policlinico.ba.it

**Keywords:** West Nile virus (WNV), Bayesian spatio-temporal Poisson model, risk mapping, climatic and environmental factors, multisource surveillance, Italy

## Abstract

West Nile virus (WNV) transmission has intensified and expanded in Italy, but quantitative evidence on how local climatic and environmental conditions influence human risk in southern regions remains limited. This study examined the association between meteorological, environmental, and host-related factors and West Nile virus (WNV) cases at the municipal level in Apulia in 2023. Human WNV cases were georeferenced at the municipal level and linked to monthly indicators (minimum and maximum temperature, precipitation, surface water extent, green area coverage, land-use change, avian occurrence). A Bayesian spatio-temporal Poisson model with a conditional autoregressive structure was fitted to monthly counts of human WNV cases, with equine WNV cases, climatic, environmental and avian indicators included as covariates. Eight human WNV cases were reported between August and October and four equine WNV cases between September and November, with partial spatial and temporal overlap. In univariable analyses, the strongest associations were observed for meteorological variables, particularly temperature. In the final multivariable model, higher maximum temperature at a two-month lag was associated with increased WNV risk (RR = 1.53; 95% CrI: 1.18–2.23), while minimum temperature was excluded due to collinearity with maximum temperature. Green area coverage and water body extent showed uncertain effects. Model-based maps indicated that elevated fitted risk was concentrated in a narrow temporal window between August and October, expanding sharply across the region in September before receding, rather than describing a stable, spatially fixed hotspot. This exploratory analysis suggests that reported human WNV infections in Apulia in 2023 were temporally concentrated during the late summer/early autumn period and that maximum temperature at a two-month lag was positively associated with the outcome in the selected model. The findings are hypothesis-generating and should be interpreted with caution given the very small number of events, but they illustrate the feasibility of integrating multisource epidemiological, climatic, environmental, and veterinary data to support locally tailored early-warning efforts in southern Italy.

## 1. Introduction

Vector-borne diseases (VBDs) are illnesses caused by parasites, viruses, or bacteria transmitted by vectors, often blood-feeding insects such as mosquitoes. Annually, VBDs result in over 700,000 deaths globally, including diseases such as malaria, dengue, chikungunya, Zika and West Nile Disease (WND) [1]. West Nile virus (WNV) has emerged as one of the most important arboviral threats in Europe, causing recurrent seasonal outbreaks and neuroinvasive disease in humans [2].

WNV is a mosquito-borne flavivirus maintained in a bird–mosquito–bird transmission cycle, with Culex pipiens mosquitoes as primary vectors and it causes WNV infection in humans [3]. Most human WNV infections are asymptomatic or pauci-symptomatic; about 20% cause mild febrile illness, and less than 1% progress to severe neuroinvasive disease, mainly in elderly or immunocompromised individuals, potentially resulting in long-term complications or death [4]. In 2023, eight human WNV notifications were recorded in Apulia, including six clinical WNND cases and two asymptomatic viremic blood donors. “Equine WNV cases” refer to laboratory-confirmed clinical infections in horses notified through the national veterinary surveillance system.

In Italy, the regions most affected by WNV infections have primarily been Emilia-Romagna and Veneto [5,6,7]. In more recent epidemic seasons, transmission has intensified and spread to broader geographical areas, with an earlier onset and a progressive expansion toward Central and Southern regions of Italy, raising growing concerns about the increasing risk of diffusion in these territories [7,8].

Following the equine outbreak that occurred in the summer of 1998 in the Tuscany Region [6], in which clinical and neuropathological features of WNV-associated equine encephalomyelitis were described [9], the Italian Ministry of Health established a national surveillance plan in 2002, which enabled the detection of WNV circulation among birds, mammals, and vectors across Emilia-Romagna, Veneto, and Lombardy. The first neuroinvasive case of West Nile disease in Italy was reported in September 2008 with an outbreak resulting in 77 infected horses and two human cases; following this event, WNV was officially declared endemic in Italy [5,6,10]; then the WNV was detected in 14 Italian regions.

The 2018 season marked a particularly severe epidemic peak. Concurrently, veterinary surveillance recorded increased WNV activity in mosquitoes, birds, and horses across 9 regions (Emilia-Romagna, Veneto, Lombardy, Sardinia, Friuli-Venezia Giulia, Piedmont, Lazio, Basilicata, and Apulia) underscoring the broad ecological diffusion of the virus and the need for integrated, nationwide monitoring strategies [8,11,12].

In Apulia, a region in South-Eastern Italy, the first locally acquired infection was reported in 2013; since then, no further cases of WNV infection—whether imported or locally acquired—have been reported. In 2023, coinciding with a Europe-wide spread of WNV infections, an unexpected outbreak and rapid increase in WNV cases were reported in the Apulia region, with eight confirmed human WNV infections, including six cases of neuroinvasive disease and two asymptomatic viremic blood donors, as well as the spread of the virus among animal hosts [13].

Climatic and environmental factors play a crucial role in shaping WNV distribution and transmission dynamics by affecting the distribution of VBD [14]. Climate change can influence pathogens, vectors and reservoir hosts by expanding geographic ranges of competent vectors and extending their seasonal activity, thereby creating more favorable conditions for WNV transmission [15,16]. Although this relationship may appear intuitive, some studies suggest that mosquito community metrics may be more informative than bird community metrics in explaining variation in WNV transmission [17,18]. Warmer temperatures can shorten the extrinsic incubation period and extend the transmission season, while land use, surface water and vegetation structure affect mosquito breeding habitats and bird communities [19]. Recent work has emphasized the importance of vector–host interfaces and microclimatic conditions in determining arboviral risk in Mediterranean and European settings [20,21].

Early warning systems (EWS) integrate entomological, veterinary, epidemiological, environmental, and climatic data to forecast outbreaks, providing advance notice that can improve the timing and targeting of vector control and public health interventions. Several modelling studies have shown that combining meteorological and land-use indicators can help predict vector dynamics or human WNV incidence, particularly in Northern Italy and other European settings [22,23,24].

Despite this growing body of evidence, quantitative analyses of how local climatic, environmental, and host-related factors are associated with WNV risk in Southern Italy remain limited. Apulia is characterized by heterogeneous landscapes, extensive agricultural areas and wetlands, and the presence of competent vectors and reservoir hosts, yet has historically reported fewer human cases than Northern regions. Understanding whether and how climatic and environmental patterns, together with equine and avian host distribution, contribute to the spatial and temporal clustering of WNV cases at the municipal level is essential to generate hypotheses for locally tailored EWS and refine regional surveillance plans [7,8].

The aim of this work was to investigate the spatio-temporal association between climatic, environmental and host-related factors, including equine WNV cases as a sentinel of local viral circulation, and the occurrence of laboratory-confirmed human WNV infections at the municipal level in the Apulia region during 2023, using a Bayesian spatio-temporal Poisson model. The outcome included eight human WNV infections, comprising six WNND cases and two asymptomatic viremic blood donors identified through routine screening.

## 2. Materials and Methods

### 2.1. Data Collection

#### 2.1.1. Hosts

Wild birds are the main natural reservoirs of WNV, and several species play an important role in virus amplification and transmission. In Italy, the common blackbird (*Turdus merula*) and the magpie (*Pica pica*) are among the preferred avian hosts of the primary vector, Culex pipiens. Other relevant species include the Eurasian jay (*Garrulus glandarius*), the hooded crow (*Corvus corone cornix*), the collared dove (*Streptopelia decaocto*), and house sparrow (*Passer domesticus*) [25,26]. The selection of monitored bird species followed national guidance on WNV avian surveillance priorities [27].

For the purposes of this study, these species were considered jointly as a composite avian host community because of their overlapping ecological niches and shared epidemiological role in WNV amplification [28]. Bird occurrence records for the six selected species were downloaded from the Global Biodiversity Information Facility (GBIF) using the rgbif R package for the period from July 2022 to December 2023 [29,30]. Occurrence records for the six selected avian species were obtained from GBIF for the period of July 2022 to December 2023. Records with available geographic coordinates were assigned to municipalities in Apulia through a point-in-polygon spatial join using GADM level-3 municipal boundaries. For each municipality and calendar month, eligible occurrence records for the six species were aggregated into a single composite occurrence-based avian host index. This variable represents the number of recorded occurrences of the selected avian host community and was not interpreted as a direct measure of absolute bird abundance. Municipality-months without eligible records were assigned a value of zero, indicating no recorded occurrence rather than confirmed absence.

To assess potential delayed associations with WNV occurrence, one- to six-month lagged versions of the monthly occurrence-based index were generated for each municipality. The use of records beginning in July 2022 allowed lagged values to be defined for all months of 2023.

#### 2.1.2. WNV Cases

West Nile virus case data for Apulia during the study period were obtained through the official national arbovirus platform [31,32]. Human WNV infections were defined as laboratory-confirmed infections identified through clinical surveillance or blood-donor screening, irrespective of clinical presentation. WNND cases were laboratory-confirmed infections with neurological involvement, including encephalitis, meningitis, or acute flaccid paralysis, according to the national surveillance case definition. In 2023, eight human WNV infections were reported in Apulia: six WNND cases and two asymptomatic viremic blood donors identified through routine screening [31,32]. Human cases were geocoded at the municipality level using the municipality recorded in the national surveillance database. The available surveillance data did not systematically provide individual-level coordinates, detailed travel histories, or confirmed probable place of exposure. Therefore, the recorded municipality could not necessarily be interpreted as the municipality in which infection was acquired. Equine case data were obtained from the national surveillance platform. The available information included the date of confirmation and municipality in which each case was reported. Equine cases were geocoded at the municipality level using this recorded municipality. Information on the probable site of exposure was not available.

#### 2.1.3. Climatic and Environmental Predictors

The following predictors were considered in the model: minimum and maximum temperature, precipitation, green area coverage, land-use change, and water body extent.

Temperature (minimum and maximum) and precipitation data were obtained from the WorldClim 2.1 monthly weather product, based on CRU TS 4.09 and bias-corrected with WorldClim 2.1, and covered the period 2020-2024 at a spatial resolution of 2.5 arcminutes. Minimum and maximum temperature were expressed in °C, while precipitation was expressed as total monthly precipitation in millimeters [33,34].

Green area coverage was derived from the Copernicus Data Space using the High-Resolution Layer Tree Cover Density (TCD) raster for the years 2022 and 2023 and aggregated at the municipal level as the mean percentage of tree canopy cover. Land-use change was approximated using the Copernicus Land Monitoring Service High Resolution Layer Imperviousness Change dataset for 2018–2021, accessed through the Copernicus Browser, and summarized at the municipal level as an indicator of soil sealing and land-use modification. Because this was the most recent dataset available for this indicator at the time of the analysis, the 2018–2021 pattern was used as a proxy for 2022–2023, assuming no major changes in the spatial distribution [35].

Water body extent was derived from the Copernicus Global Land Service Water Bodies product using the monthly rasters available for 2022 and 2023 and aggregated at the municipal level as an indicator of surface water extent [36].

All spatial datasets were downloaded and subsequently cropped and masked to the boundaries of the study region prior to analysis. To reconcile the high spatial resolution of the raster layers with the coarser administrative units used by the national surveillance systems, all predictors were summarized at the municipal level. This approach allowed us to integrate human WNV case counts with the corresponding environmental and climatic characteristics at the same scale.

### 2.2. Data Analysis

#### 2.2.1. Model Specification

To evaluate the association between environmental and ecological factors and WNV risk, a Bayesian spatio-temporal model was implemented using the CARBayesST package in R software [37]. The outcome was modelled using a Poisson regression framework, with the log of the 2023 resident municipal population included as an offset. To account for spatial autocorrelation among contiguous municipalities and temporal autocorrelation across consecutive months, a Bayesian conditional autoregressive (CAR) model with a first-order autoregressive temporal structure was applied via the ST.CARar() function, allowing the spatio-temporal structure of the data to be explicitly modelled [37].

Let $Y_{it}$ denote the number of confirmed human WNV infections observed in municipality $i\;\left(i=1,\ldots ,I\right)$ during month $t\;\left(t=1,\ldots ,T\right)$. Conditional on the expected number of infections, $\mu_{it}$, the outcome was modelled as

(1) $${Y_{it}|\mu }_{it}∼\mathrm{Poisson}\left(\mu_{it}\right)$$

The logarithm of the expected number of human WNV infections was modelled as

(2) $$\log\left(\mu_{it}\right)=\log\left(P_{i}\right)+\beta_{0}+X_{it}^{⊤}\beta +\phi_{it},$$

where $P_{i}$ is the 2023 resident population of municipality i, included as a log-offset; $\beta_{0}$ is the intercept; $X_{it}^{⊤}$ is the vector of municipality- and month-specific covariates; β is the vector of regression coefficients; $\phi_{it}$ is the residual spatio-temporal random effect. Thus, the model estimates associations with the expected monthly incidence of human WNV infection while accounting for differences in municipal population size.

The outcome included all eight laboratory-confirmed human WNV infections recorded in Apulia in 2023, comprising six WNND cases and two asymptomatic viremic blood donors. Equine WNV cases were included as a sentinel covariate in selected model specifications and were not part of the outcome. Let:

(3) $$\phi_{t}={\left(\phi_{1t},\ldots ,\phi_{Kt}\right)}^{⊤},$$

denote the vector of residual spatio-temporal random effects across the K municipalities at month t. The random effects were modelled using a first-order autoregressive multivariate conditional autoregressive process:

(4) $$\phi_{t}|\phi_{t-1}∼N\left(\rho_{T}\phi_{t-1},\tau^{2}Q{\left(W,\rho_{S}\right)}^{-1}\right),\;\;t=2,\ldots ,T,$$

The spatial precision matrix was defined according to the Leroux CAR formulation:

(5) $$Q\left(W,\rho_{S}\right)=\rho_{S}\left(D-W\right)+\left(1-\rho_{S}\right)I,$$

where W is the binary municipal adjacency matrix, D is the diagonal matrix of the number of neighbors for each municipality, and I the identity matrix. The parameter $\rho_{S}$ quantifies residual spatial autocorrelation, whereas $\rho_{T}$ describes first-order temporal dependence between consecutive months. The parameter $\tau^{2}$ represents the conditional variance of the residual spatio-temporal process.

Gaussian priors were assigned to the intercept and regression coefficients:

(6) $$\beta_{p}∼N\left(0,100,000\right),$$

whereas Uniform $\left(0,1\right)$ priors were assigned to the spatial and temporal autocorrelation parameters:

(7) $$\rho_{S},\rho_{T}∼Uniform\left(0,1\right)$$

The variance parameter was assigned the default inverse-gamma prior implemented in CARBayesST:

(8) $$\tau^{2}∼Inverse\text{-}Gamma\left(1,0.01\right).$$

Spatial adjacency was defined using queen contiguity between municipal polygons, such that municipalities sharing either a boundary segment or a vertex were considered neighbors. Municipal boundaries were obtained from the GADM level-3 dataset, converted to valid geometries, and projected to WGS84/UTM zone 33N (EPSG:32633) before adjacency calculation. A snap tolerance of 10 m was applied to account for minor topological gaps between polygon boundaries. The initial queen-contiguity neighbor list included one municipality with no first-order neighbors. To avoid an isolated node in the CAR spatial graph, this municipality was connected to the geographically nearest municipality on the basis of centroid-to-centroid distance. The neighbor list was then symmetrized and converted into a binary, unweighted adjacency matrix. The final matrix included 258 municipalities and 684 undirected neighbor links; it was symmetric, had a zero diagonal, included no isolated municipalities, and formed a single connected component. Each municipality had between one and 15 neighbors. The order of municipalities in the adjacency matrix was verified to correspond exactly to the municipality order in each monthly block of the modelling dataset.

The data were ordered by month and municipality, such that all municipalities were listed within each monthly block, as required by the ST.CARar() implementation [37].

Bayesian inference was performed using Markov chain Monte Carlo (MCMC) simulation. Each model was run for 500,000 iterations with a burn-in of 50,000 iterations and a thinning interval of 5 across three independent chains. Convergence was assessed using Gelman–Rubin potential scale reduction factors (PSRF) and effective sample sizes (ESS). We considered PSRF values below 1.10 as a general convergence target and aimed for values below 1.05 for fixed-effect coefficients. For the selected exploratory model, fixed-effect coefficients had PSRF values close to 1, whereas the latent spatio-temporal variance parameter showed slower mixing (PSRF approximately 1.3). Parameter-specific PSRF and ESS values are reported in Table 1 and Supplementary Tables S2A,B and S3A,B.

Model development followed a hierarchical, biologically informed strategy rather than an unrestricted comparison of all possible predictor combinations. Each candidate predictor, including equine WNV cases as a sentinel covariate, was first evaluated in a univariable spatio-temporal model. For climatic variables, lag periods of 1–6 months were examined within a biologically plausible window reflecting the mosquito life cycle and the expected delay between environmental conditions and WNV transmission; minimum and maximum temperature at 1- and 2-month lags, which showed the strongest and most consistent associations in the univariable screening, were carried forward as core covariates in subsequent multivariable models. The remaining environmental and ecological variables (precipitation at lag 2, water body extent, land-use change, green area coverage, avian occurrences reported) were then combined in multivariable specifications as described in Section 2.2.2 and Section 2.2.3.

#### 2.2.2. Lag Selection

Temperature predictors were evaluated at multiple time lags, from one to six months prior to case onset, within the biologically plausible window defined above. In the univariable screening, one- and two-month lags showed the strongest and most consistent associations with the outcome, whereas longer lags (three to six months) were weaker and less consistent and were therefore not retained. Both the one- and two-month temperature lag specifications were initially evaluated in parallel in multivariable models; in the preliminary non-spatial Poisson GLM screening, models including either the 1-month or the 2-month temperature lag pair showed very similar regression estimates and comparable AIC and BIC values, indicating no clear advantage of one lag structure over the other in terms of statistical fit. On this basis and given the stronger biological plausibility of a 2-month lag in relation to the expected delay between environmental exposure, mosquito abundance, vector competence, and WNV transmission, the 2-month temperature lag was retained as the reference structure for all subsequent spatio-temporal models.

#### 2.2.3. Model Selection

Model selection was performed within the lag-2 framework. All combinations of the candidate predictors were fitted using a Poisson generalized linear model—that is, a model without spatial or temporal correlation structure—and the Variance Inflation Factor (VIF) was computed for each combination to assess multicollinearity. Only combinations with a maximum VIF below the prespecified threshold of 5 were retained as admissible. VIF was used exclusively as a collinearity-screening criterion, rather than as a variable-selection criterion. The choice of covariates to combine was guided by biological plausibility and included the temperature core, precipitation at lag 2, water body extent, land-use change, green area coverage, occurrence-based avian host index, and equine WNV cases.

Given the planned role of equine WNV cases as a contextual sentinel covariate of local viral circulation, model building was structured into two complementary sets of specifications. The first set comprised primary spatio-temporal models in which equine cases were retained as a fixed sentinel covariate together with the lag-2 temperature core and, in different specifications, one additional environmental or ecological covariate (precipitation, water body extent, land-use change, green area coverage, or the occurrence-based avian host index), with and without minimum temperature. Among the admissible specifications, seven biologically motivated models were selected for detailed Bayesian evaluation. These models were intended to examine a limited set of plausible covariate structures rather than to identify a definitively optimal model. Models were compared using DIC and WAIC, together with LMPL as a complementary model-comparison measure, parameter-specific convergence diagnostics, effective sample sizes, biological interpretability, and parsimony. No formal composite score was used. Full results for these seven primary models, including covariate structure, model-comparison indices, complexity measures, and MCMC convergence diagnostics, are reported in Tables S2A–S3A of the Supplementary Material. Supplementary Table S4 provides a focused comparison of the final primary model specifications considered in selecting the exploratory model used for inference and mapping.

The second set consisted of an extended sensitivity analysis restricted to environmental and ecological covariates alone (precipitation, water body extent, land-use change, green area coverage, and the occurrence-based avian host index), fitted together with the lag-2 temperature core but excluding equine cases. This second set comprised all 26 admissible combinations of two to five covariates, subject to VIF < 5. It was designed to examine whether the estimated associations of environmental and ecological predictors changed when the equine sentinel covariate was excluded from the model. This approach allowed the contribution of climatic, environmental, and host-related predictors to be explored both in the presence and absence of the equine covariate, without treating the sensitivity models as equivalent candidates in the selection of the primary model. Full results for these 26 sensitivity models are reported in Tables S2B–S3B of the Supplementary Material.

#### 2.2.4. Spatial Risk Mapping

Model-fitted values from the final Bayesian spatio-temporal model were used to derive municipality-level risk indices for each month of the year 2023. For each municipality and month, the fitted number of human WNV infections was extracted and linked to the corresponding municipal geometry using the unique GADM identifier (Database of Global Administrative Areas; GID_3, i.e., the unique identifier for third-level administrative units, corresponding here to municipalities), allowing the spatial visualization of the estimated disease burden across Apulia.

Municipality- and month-specific fitted incidence rates were calculated by dividing the model-fitted case counts by the corresponding municipal population. The regional average monthly incidence rate over 2023 was used as a common reference and was calculated as the total fitted number of human WNV infections divided by 47,003,148 person-months, corresponding to 3,916,929 residents followed over 12 months. The area-level incidence rate ratio (IRR) for municipality i in month t was defined as:

(9) IRRit=μˆit(Ni ×1 month)∑i∑tμˆit/(Ntot×12 months)

where ${\hat{\mu }}_{it}$ denotes the model-fitted number of human WNV infections in municipality i during month t, $N_{i}$ is the resident population of municipality i, and $N_{tot}$ is the total regional population. Values greater than 1 indicate a fitted municipal monthly incidence above the regional annual-average monthly reference, whereas values below 1 indicate a fitted incidence below this reference. A common annual-average monthly reference was used across all maps to permit direct comparison of fitted relative-incidence levels between months. For cartographic representation, IRR values were categorized into five classes: [0,1),[1,1.5),[1.5,3),[3,5), ≥5. Maps were produced for each of the 12 months, both as individual figures and as a single faceted panel allowing visual inspection of the spatio-temporal evolution of risk across the year 2023. These maps display spatially and temporally smoothed model-fitted relative-incidence surfaces, with observed human WNV infections overlaid as white circles with dark outlines. The fitted surfaces should be interpreted separately from the observed case markers and should not be regarded as direct evidence of local WNV transmission in each municipality-month. Elevated fitted values in municipality-months without observed infections may arise from the spatial and temporal dependence structures borrowing information across neighboring municipalities and adjacent months, as well as from the included covariates; such values should therefore not be interpreted as evidence of local transmission. As a supplementary analysis, we calculated a month-specific municipality-to-region model-based $IRR_{it\_monthly}$ by dividing the fitted municipal incidence by the fitted regional incidence in the same calendar month. Where posterior fitted-value samples were available, posterior median IRRs, 95% credible intervals, and exceedance probabilities were derived. These analyses assessed the robustness of spatial contrasts to the temporal reference and quantified posterior uncertainty. Posterior median IRR maps and maps of Pr($IRR_{it\_monthly}>1$) are presented in Figures S1 and S2, respectively.

## 3. Results

### 3.1. Descriptive Analysis

**Figure 1:**
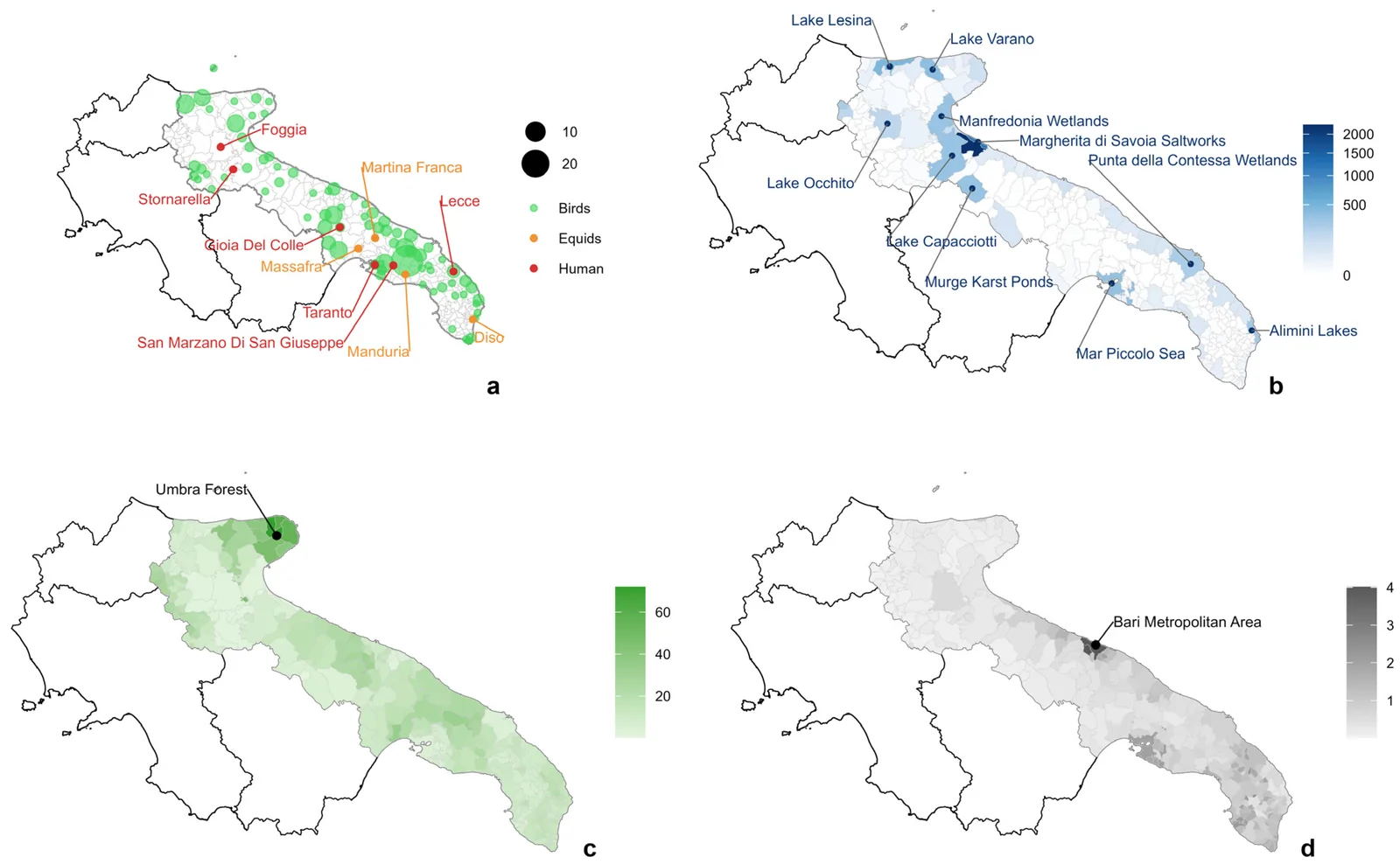
Spatial distribution in Apulia in 2023 of (**a**) birds (green), human cases (red), and equine cases (orange); (**b**) water body extent (blue), with the main lakes and wetlands labeled; (**c**) annual mean green area coverage (graduated green), with Umbra Forest highlighted; and (**d**) land-use change (grey), based on the 2018–2021 dataset, the most recent available for this indicator, with the Bari Metropolitan Area labeled.

In 2023, a total of eight human West Nile virus (WNV) notifications were reported in Apulia: six clinical West Nile neuroinvasive disease (WNND) cases and two asymptomatic viremic blood donors identified through screening. These were temporally distributed as follows: two cases occurred in August in the province of Foggia, three cases were reported in September, with two in the center of the region (Bari) and one in the southern part (Lecce), and three cases occurred in October, in the south-western part of the region (Taranto). Four equine WNV cases showed a partially overlapping distribution, occurring from September to November, with three cases in Taranto and one in Lecce. The distribution of reported birds was largely uniform across the region (Figure 1a).

The distribution of wet areas (Figure 1b), estimated as a municipality-level measure of surface water extent derived from the Copernicus Water body extent 100 m monthly product, showed a limited extent of surface water presence across the region. Only a few areas in Apulia presented a substantial water body extent: the most prominent feature on the map was the Margherita di Savoia Saltworks, followed by smaller but locally relevant water body extent including Lake Lesina, Lake Varano, the Alimini Lakes, the Manfredonia Wetlands, Mar Piccolo Sea, Lake Capacciotti, the Murge Karst Ponds, the Punta della Contessa Wetlands, and Lake Occhito, most of which are only marginally visible at the scale of the figure.

Green areas (Figure 1c), expressed as the mean percentage of tree and forest cover per municipality, appeared to be uniformly distributed across the whole region, with higher density in the northern area, particularly in the Gargano promontory, where the Umbra Forest represents the area of highest green coverage in Apulia.

Land use changes between 2018 and 2021 (Figure 1d), expressed as the percentage of municipal area affected by land-cover modification and representing the most recent data available for this indicator, showed that land-cover change was more pronounced in the major urban areas. The highest density was observed in the six provincial capitals: from north to south, Foggia, Barletta, Bari (in the western part), Taranto, Brindisi (in the eastern part), and Lecce, with the Bari Metropolitan Area showing the most extensive change overall.

**Figure 2:**
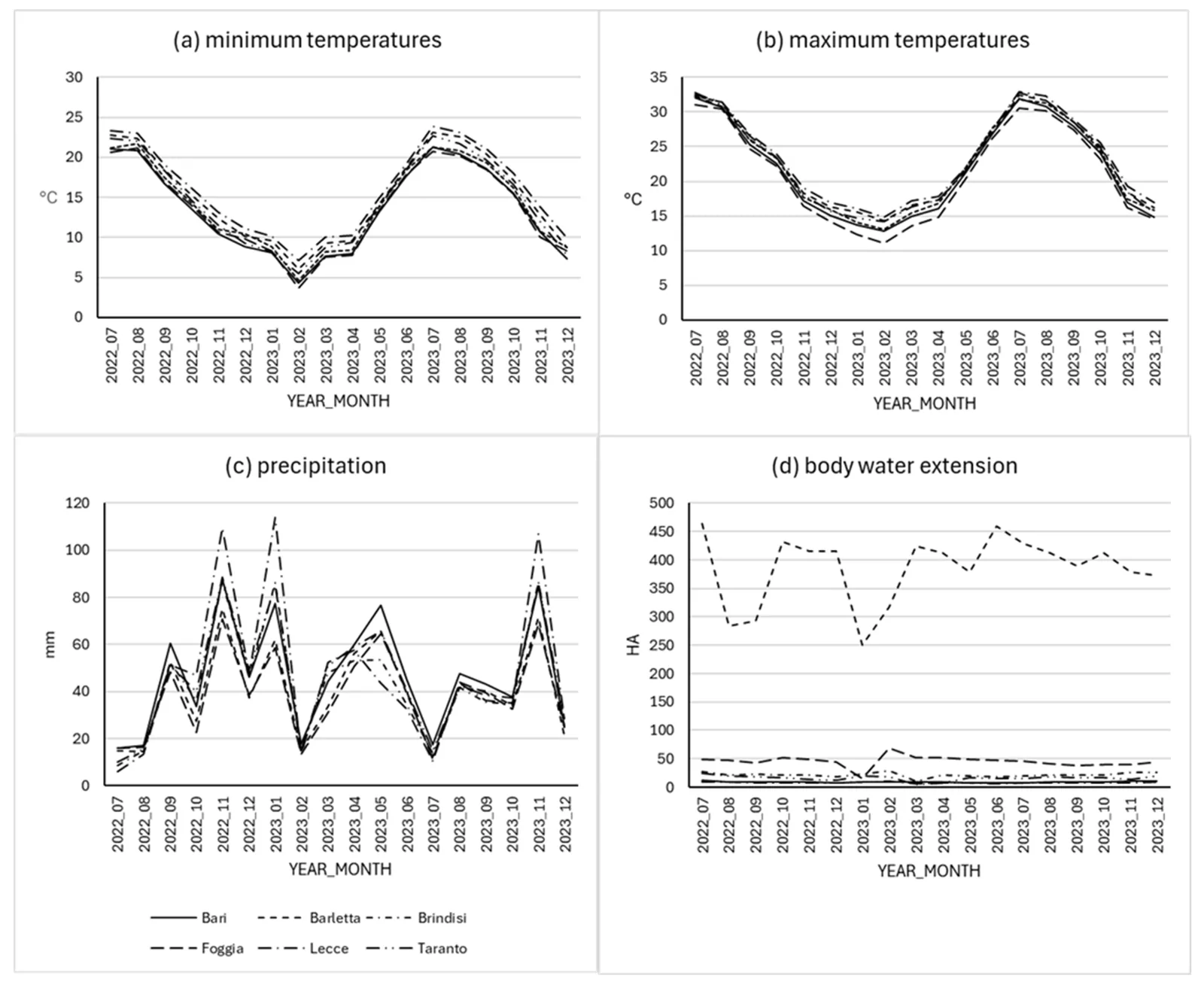
Temporal trends in minimum temperatures (**a**) maximum temperature (**b**), precipitation (**c**) and water body extension (**d**) from July 2022, corresponding to six-month lag for January 2023, to December 2023, the final month included in the analysis of human WNV incidence.

The temporal trends in temperature (both minimum and maximum), precipitation and water body extent, did not show substantial differences across municipalities and provinces. However, temperature exhibited a clear seasonal pattern: both minimum and maximum temperatures increased from May in the years considered, peaked in August, and gradually decreased in the following months (Figure 2a,b). Precipitation showed greater variability over time, with lower values from March 2023 to October 2023, higher levels observed in November of both years 2022 and 2023. No clear differences were observed across municipalities or provinces (Figure 2c). Water bodies showed a limited extension of wet areas in the region, with values that appeared relatively constant over time and similar across geographic areas, except for the province of Barletta (Figure 2d). This difference may be explained by the presence of the Margherita di Savoia salt pans along the coastline.

### 3.2. Univariate Analysis

In the univariable spatio-temporal models, the clearest associations were observed for meteorological variables and equine WNV cases. Equine cases, included as a contextual sentinel covariate, showed a positive but highly imprecise association with human WNV incidence, with a very large posterior mean incidence rate ratio but wide credible intervals (RR = 36, 95% CrI: 1.6–291), reflecting sparse data and substantial uncertainty about the exact magnitude of the effect. Precipitation at a two-month lag was inversely associated with WNV risk (RR = 0.97, 95% CrI: 0.93–1.00). Both maximum and minimum temperature were positively associated with WNV risk from the contemporaneous month up to lag 3, with the strongest effects at lag 2 (maximum temperature: RR = 1.59, 95% CrI: 1.22–2.33; minimum temperature: RR = 1.48, 95% CrI: 1.18–2.01). At lag 6, this pattern reversed, with inverse associations for both maximum and minimum temperature (maximum temperature: RR = 0.77, 95% CrI: 0.61–0.93; minimum temperature: RR = 0.72, 95% CrI: 0.57–0.88), indicating inverse associations between temperature and WNV risk at longer lags. Land-use change, water body extent, green area coverage, and occurrence-based avian host indices showed weaker and more uncertain associations, with posterior credible intervals generally including the null value. Full univariable results for all covariates are reported in Table S1 of the Supplementary Material.

### 3.3. Multivariable Analysis

In the selected multivariable spatio-temporal Poisson model (CAR_lag2_7) with equine cases as a sentinel covariate and without minimum temperature (Table 1), maximum temperature at a two-month lag was positively associated with the expected incidence of human WNV, with an estimated RR of 1.53 (95% CrI: 1.18–2.23). The equine WNV covariate showed a positive association (RR = 20.88; 95% CrI: 0.74–277.41), but this estimate was highly imprecise and should not be interpreted as confirmation of an independent sentinel effect. Green area coverage and water body extent displayed RRs close to the null (RR = 0.92, 95% CrI: 0.81–1.03, and RR = 1.00, 95% CrI: 0.99–1.00, respectively), indicating no clear evidence of an association at the spatial and temporal resolution considered. The estimated spatial (ρS= 0.40, 95% CrI: 0.02–0.90) and temporal (ρT= 0.36, 95% CrI: 0.01–0.88) autocorrelation parameters indicated moderate residual dependence in both space and time, though with wide credible intervals reflecting substantial uncertainty. The latent variance parameter ($\tau^{2}$ = 0.011, 95% CrI: 0.002–0.043) suggested the presence of residual unexplained spatio-temporal variability. Regression coefficients showed PSRF values close to 1 and adequate effective sample sizes. The spatial and temporal dependence parameters showed slower mixing, and the latent variance parameter had a PSRF of 1.3, indicating incomplete convergence of this component. Therefore, estimates from the selected model should be interpreted cautiously as exploratory. Model-comparison indices and convergence diagnostics for the selected exploratory model and the principal competing specifications are summarized in Supplementary Table S4, which also reports parameter-specific convergence summaries for the principal competing models, including slower mixing for selected latent variance components.

Based on the selected multivariable spatio-temporal model, maximum temperature at a two-month lag was associated with expected monthly human WNV incidence (RR = 1.53; 95% CrI: 1.18–2.23). This association should be interpreted as exploratory evidence under a linear model specification rather than as evidence of a specific temperature threshold, as neither nonlinear nor change-point structures were evaluated, and the number of observed events was very small.

**Figure 3:**
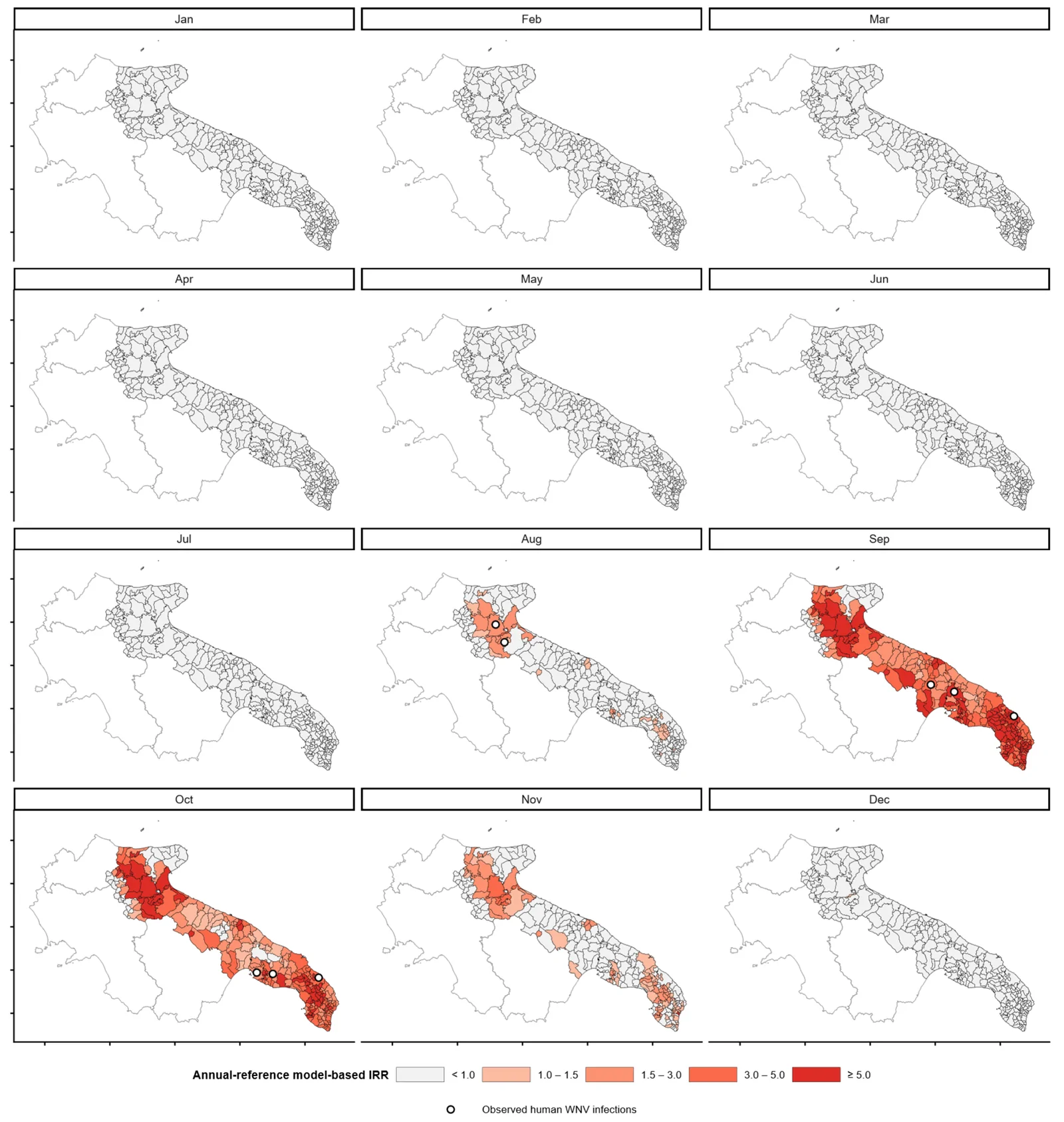
Monthly spatial distribution of the annual-reference model-based incidence rate ratio (IRR) for human West Nile virus (WNV) infections across Apulian municipalities in 2023. The IRR compares fitted municipal monthly incidence with the Apulia-wide average monthly fitted incidence over the study year; values above 1 indicate fitted municipal incidence above this reference. Colored areas represent spatially and temporally smoothed model-fitted relative-incidence estimates, whereas white circles with black outlines indicate municipalities and months with observed human WNV cases.

Figure 3 shows the monthly spatial distribution of the fitted incidence rate ratios (IRRs) for human WNV infections across Apulian municipalities, estimated by the final Bayesian spatio-temporal model, which included equine WNV cases as a sentinel covariate and maximum temperature at a two-month lag. The IRR compares the fitted municipal monthly incidence with the Apulia-wide average monthly fitted incidence over 2023.

From January to July, fitted IRR values were below 1.0 across virtually the entire region. A localized increase first appeared in August, when IRR values in the 1.0–3.0 range emerged in a limited number of municipalities in northern Apulia particularly in the province of Foggia. The most pronounced fitted elevation occurred in September, when municipalities spanning the whole region—from the north, through the central provinces, down to the Salento peninsula—showed IRR values in the 3.0–5.0 and ≥5.0 classes.

In October, elevated IRR values persisted, with a spatial pattern that concentrated more markedly in the western and southern parts of the region (Taranto and the Salento peninsula), while November showed a marked contraction, with only a few municipalities in the northwest and southeast retaining IRR values above 1.0. By December, IRR values returned to below 1.0 across the entire region.

Overall, the fitted relative-incidence surface was strongly time-dependent, with a sharp increase in September and October followed by a subsequent decline. This pattern does not support the interpretation of a stable, geographically fixed hotspot. Given the small number of human infections and the spatial-temporal smoothing intrinsic to the model, the mapped pattern should be interpreted as exploratory and hypothesis-generating rather than as direct evidence of local transmission in each municipality-month.

Supplementary analyses using a month-specific municipality-to-region reference indicated spatial heterogeneity in model-estimated relative incidence across Apulian municipalities. Areas with posterior median IRR values above 1 were observed in several months, although the geographical distribution and the posterior support for IRR >1 varied over time. The corresponding posterior median IRR maps and posterior exceedance-probability maps are reported in Supplementary Figures S1 and S2, respectively. These results should be interpreted as exploratory model-derived spatial contrasts rather than direct evidence of local WNV transmission in individual municipality-months.

## 4. Discussion

This study presents a municipal-level, Bayesian spatio-temporal analysis of WNV risk in Apulia during the 2023 transmission season, integrating human WNV notifications and equine WNV cases with climatic and environmental indicators at the municipal level. Consistent with the epidemiology of WNV in Italy, human WNV notifications in Apulia were concentrated between August and October, with two cases in August and three cases in both September and October. Notifications were geographically clustered in Taranto (*n* = 3), Foggia (*n* = 2), Lecce (*n* = 2), and Bari (*n* = 1), with no human WNV cases reported in Barletta-Andria-Trani or Brindisi, and equine WNV cases showed a similar spatial and temporal distribution. This temporal pattern mirrors findings from Northern Italy, where the intensity of human transmission has been shown to follow the build-up of infection in mosquito and avian reservoirs during the warm months [38]. The partial temporal and spatial overlap between human and equine notifications is consistent with the potential value of considering veterinary information alongside human surveillance, while not establishing a causal or prospectively predictive role for equine cases in this dataset [2].

In univariable models, meteorological variables—particularly maximum and minimum temperature—showed the clearest temporal patterns. Both maximum and minimum temperature were positively associated with human WNV infections from the contemporaneous month up to lag 3, with the strongest effects at lag 2, where RR reached 1.59 for maximum temperature and 1.48 for minimum temperature. At lag 6, the direction of the association reversed for both indicators, with RR below 1 for maximum and minimum temperature, a pattern that has also been reported in previous studies [38]. Precipitation showed a weaker but still informative lag structure, with a protective association at lag 2 and estimates closer to the null at other lag times. By contrast, land-use change, water body extent, and avian occurrences showed weaker and more uncertain associations in the univariate screening phase. These results are broadly coherent with the biological role of temperature in mosquito development, biting activity, and viral amplification, but they do not establish a causal temperature–incidence relationship.

In the selected exploratory multivariable model with equine cases as a sentinel covariate, maximum temperature at a two-month lag showed the strongest positive association with human WNV incidence (RR = 1.53; 95% CrI: 1.18–2.23). Although biologically consistent with the expected ecology of WNV transmission, this estimate should be interpreted in light of the very limited number of events and the uncertainty introduced by evaluating alternative lag and model specifications, and it cannot be taken as evidence of a specific temperature threshold. This result suggests that conditions occurring several weeks before human onset may be more relevant than more distant climatic patterns. Minimum temperature was not retained in the final model, and the sensitivity analyses suggested substantial overlap between maximum and minimum temperature during the warm months, with limited independent variation in minimum temperature once maximum temperature was included, consistent with a possible collinearity between the two indicators rather than a distinct effect of minimum temperature. Green area coverage and water body extent also remained in the final model, but their estimates were close to null and compatible with substantial uncertainty. Taken together, these results suggest that, at the spatial and temporal resolution used here, within the selected model, dynamic meteorological conditions showed a more apparent signal than static environmental indicators, although this finding requires confirmation in larger, multi-year datasets [7,24].

Most environmental and ecological covariates showed imprecise and model-dependent associations. Residual correlation among retained predictors may remain despite VIF-based screening, while municipal-level aggregation may obscure local heterogeneity in mosquito breeding habitats, irrigation, wetlands, host occurrence, and microclimatic conditions. In addition, the occurrence-based avian host index and the equine sentinel covariate are subject to heterogeneous observation and reporting processes. Together with the small number of human infections, these limitations reduce the ability to detect moderate independent associations. Consequently, estimates compatible with the null should not be interpreted as evidence that these factors are biologically irrelevant in Apulia. Larger multi-year datasets with finer-scale environmental measures and standardized entomological and ornithological surveillance are needed to assess these associations more reliably. The fitted IRR maps provide a model-based description of the estimated temporal and spatial pattern. The monthly maps show that elevated fitted risk emerged only from August onward, expanded sharply across the region in September, remained elevated—though more spatially concentrated in the west and south—in October, and then rapidly receded through November and December, with virtually no municipality showing IRR above 1.0 outside this narrow window. This temporal pattern is compatible with the observed concentration of human WNV notifications between August and October. Although some municipalities with elevated fitted IRR during the late-summer transmission peak are located in areas characterized by extensive agriculture and coastal or inland wetlands, our spatio-temporal models were fitted at the municipal level using generic land-use and environmental indicators and do not explicitly capture local olive groves, irrigation schemes, or human movement. For this reason, we deliberately avoid attributing the observed patterns of elevated fitted risk to specific ecological or socioeconomic processes and instead interpret them as model-based signals of where and when WNV risk may transiently increase, requiring further investigation. This interpretation is also consistent with the residual spatial and temporal dependence estimated in the final model. The spatial autocorrelation parameter and the temporal autocorrelation parameter both indicated moderate residual dependence, although with wide credible intervals. This suggests that part of the variation in WNV risk was still structured in space and time even after accounting for the observed covariates, and may reflect the contribution of unmeasured ecological, entomological, or host-related determinants [38,39,40]. Thus, the mapped pattern is better interpreted as the monthly distribution of model-estimated relative risk, highlighting a sharply time-bound period of elevated risk concentrated in September–October, rather than evidence of a persistent, geographically fixed hotspot. The mapped annual-reference IRR and supplementary month-specific municipality-to-region IRR estimates should be interpreted as exploratory, spatially and temporally smoothed model-derived contrasts. They may help generate hypotheses for future surveillance and data collection, but do not constitute evidence of persistent or sharply bounded hotspots, local transmission in individual municipality-months, or the role of specific agricultural systems in shaping WNV risk.

The modelling approach adopted in this study was a Bayesian spatio-temporal Poisson model with conditional autoregressive structure. This is in line with recent work applying Bayesian hierarchical models to WNV and other arboviral infections to account for spatial and temporal autocorrelation and to borrow strength across neighboring units [22,41]. The hierarchical framework allowed spatial and temporal dependence to be represented and information to be shared across neighboring units. However, such borrowing can stabilize estimates but cannot compensate for the limited information provided by only eight laboratory-confirmed human WNV infections. Consequently, both covariate effects and random effect estimates remain subject to substantial uncertainty.

This study has several important limitations. First and foremost, the analysis of human WNV incidence was based on only eight laboratory-confirmed human WNV infections recorded during a single transmission season and the vast majority of municipality-month observations contained no events. This severely limited the information available for estimating covariate effects and spatial and temporal components. Although the Bayesian CAR framework allowed spatial borrowing of information, it could not overcome the fundamental scarcity of events. Model development was biologically constrained by evaluating climatic lags within a plausible six-month window and by excluding multivariable specifications affected by substantial collinearity using a VIF threshold of 5. Nevertheless, the evaluation of alternative lag and model specifications introduced model-selection uncertainty that is not reflected in the credible intervals conditional on the selected model. The resulting estimates should therefore be regarded as exploratory and hypothesis-generating.

Second, aggregation of predictors at the municipal level may have attenuated finer-scale ecological relationships relevant to mosquito breeding and WNV amplification. The spatial weights matrix required one nearest-neighbor connection for a municipality without first-order queen-contiguity neighbors; although this represents a modelling choice, it affected only one of the 258 municipalities. A further limitation concerns the geographical allocation of human WNV cases. Cases were geocoded at the municipality level using the municipality recorded in the national surveillance database; however, the available data did not systematically provide the probable place of exposure, individual-level coordinates, or detailed travel histories. The recorded municipality may therefore not coincide with the location where infection was acquired. Spatial misclassification cannot be excluded and is particularly relevant given the small number of observed human cases, as the reassignment of one or two events could affect the magnitude and spatial extent of model-fitted relative-incidence contrasts. This limitation further supports a cautious interpretation of the mapped estimates as exploratory, spatially and temporally smoothed model-derived patterns, rather than definitive evidence of sharply bounded high-risk areas or persistent local transmission.

A further limitation concerns the composite avian host index derived from GBIF occurrence records. The index was based on monthly counts of georeferenced records aggregated across selected avian species within each municipality and should be interpreted as an occurrence-based proxy of recorded host presence and reporting intensity, rather than as a direct estimate of bird abundance. GBIF data are opportunistic and may be influenced by heterogeneous observation effort across space and time, including differences in observer distribution, accessibility, human population density, seasonal recording activity, taxonomic detectability, and potential duplicate reporting. Therefore, a high number of records in a municipality-month may reflect greater reporting effort as well as greater host occurrence, whereas zero records do not establish true absence. The lagged variables inherit the same limitations. Consequently, associations involving this indicator should be regarded as exploratory. Future studies should validate the occurrence-based index against standardized ornithological surveys and, where possible, account for observation effort using sampling-event data, checklists, unique observers, or total records per municipality-month.

Fourth, equine WNV cases were used as a covariate to capture local viral circulation, but differences in surveillance sensitivity and reporting probability between human and veterinary systems may introduce additional heterogeneity and potential measurement error in this proxy. Because the estimated association between equine WNV cases and human WNV incidence was highly imprecise and may vary across specifications, inclusion of this covariate may influence the estimated associations of other environmental and ecological predictors. To explore this possibility, a complementary set of sensitivity models excluding equine cases was fitted; however, this comparison remains indirect and cannot fully disentangle host-related and environmental drivers with the available data.

Finally, the limited number of events precluded a meaningful division of the data into training and validation sets. Consequently, the selected model should not be considered a validated predictive model. Its reproducibility and the appropriateness of the chosen lag structure will need to be assessed through temporal validation using subsequent surveillance years, treating the current lag specification as fixed rather than optimized in future data.

Despite these constraints, the study illustrates the feasibility of integrating multisource epidemiological, climatic, environmental, and host-related data within a Bayesian spatio-temporal framework at the subnational level. The association observed for maximum temperature at a two-month lag and the model-derived spatial contrasts observed during the late summer and autumn period provide testable hypotheses for subsequent transmission seasons rather than confirmatory evidence of stable determinants or permanent hotspots. Across several univariable and multivariable specifications considered, maximum temperature at a two-month lag showed a positive association with the expected monthly incidence of human WNV infections. However, its magnitude remained conditional on model choice and uncertain because of the small number of events. Therefore, the present analysis cannot identify a genuine temperature threshold for WNV transmission. The observed link between short-term temperature and WNV risk in Apulia is consistent with studies relating climatic factors to *Culex pipiens* population dynamics and WNV transmission in north-western Italy [42,43,44]. More generally, elevated model-fitted relative-incidence estimates in some municipalities during the late summer and autumn period suggest that future surveillance should consider both temporal seasonality and local ecological contexts in which risk may transiently concentrate. Future surveillance frameworks could evaluate the added value of veterinary and standardized ornithological information alongside climatic and entomological data, within a One Health and EcoHealth framework integrating human, animal, vector, and environmental information [45]. Furthermore, the experience gained in Apulia complements the growing body of evidence linking climate change-related factors— such as prolonged warm seasons, agricultural land use and altered hydrological regimes— to the expansion and intensification of WNV transmission in Italy [7,15,16,38,39].

Future research should build on these exploratory findings by analyzing multiple transmission seasons and incorporating veterinary and entomological surveillance data into an integrated Bayesian framework, possibly including mechanistic components that account for vector and host dynamics or additional finer-scale environmental measures. As data from subsequent transmission seasons become available, the model specification identified in this exploratory analysis should be applied without repeating the selection process, thereby allowing temporal validation of its calibration, predictive performance, lag structure, and spatial risk pattern. A multi-year framework would permit more stable estimation of lag effects and a more robust evaluation of how temperature and other climatic drivers shape WNV risk in Southern Italy. Our findings are best viewed as hypothesis-generating. The positive association between two-month lagged maximum temperature and human WNV incidence in the selected model warrants prospective assessment in subsequent transmission seasons. In contrast with findings from higher-incidence northern Italian settings, including Emilia-Romagna, associations with the occurrence-based avian host index and static environmental indicators were uncertain in the present analysis. This may reflect regional eco-epidemiological variation, but it may also result from the single-season design, limited statistical power, municipal-level aggregation, and heterogeneous observation effort in GBIF data. Future studies combining larger multi-year datasets, finer-scale land-use and irrigation information, and entomological or ornithological data will be needed to test whether these regional differences in apparent risk factors reflect genuine variation in WNV eco-epidemiology or are mainly due to data and power limitations. Such datasets will also be needed to evaluate potential nonlinear temperature–incidence relationships and to assess whether any temperature-based surveillance threshold can be identified and externally validated. In addition, linking spatio-temporal statistical models with climate change scenarios may help anticipate how WNV risk in Apulia and other Southern regions could evolve under future warming, providing actionable information for long-term public health planning [7].

## 5. Conclusions

In this exploratory analysis, maximum temperature at a two-month lag showed the strongest positive association with human WNV infection occurrence in the selected model, whereas the associations for static environmental indicators were weaker and more uncertain. Given the small number of events and the evaluation of alternative lag and model specifications, these findings should be considered hypothesis-generating and require temporal validation using data from subsequent transmission seasons.

The results do not establish a definitive hierarchy of climatic, environmental, or host-related determinants of WNV risk. Equine WNV cases were included as a contextual sentinel covariate. Their estimated association with human WNV infections was highly imprecise and should not be interpreted as confirmation of an independent sentinel effect or of prospective predictive utility.

Given the very small number of human WNV infections and the evaluation of alternative lag and model specifications, the findings should be regarded as exploratory and hypothesis-generating rather than confirmatory. Rather than providing an operational early-warning system, this study illustrates the feasibility of combining climatic, veterinary, and epidemiological information within a Bayesian spatio-temporal framework to explore the spatial and temporal distribution of human WNV infections at the municipal level. Future studies using multi-year data, standardized entomological and ornithological surveillance, and independent temporal validation will be required before model-derived indicators can be considered for locally tailored early-warning applications.

## Figures and Tables

**Table 1:** Parameters of the final Bayesian model, including 95% credible interval and Potential Scale Reduction Factors (PSRF).

Parameter	RR	95% CrI for RR	ESS	PSRF
Maximum temperature (lag 2)	1.53	1.18 to 2.23	4863.0	1
Water body extent	1.00	0.99 to 1.00	4965.9	1
Green area coverage	0.92	0.81 to 1.02	24,792.3	1
Equine WNV cases	20.88	0.74 to 277.41	8252.7	1
**Parameter**	**Posterior mean (β)**	**95% CrI**	**ESS**	**PSRF**
Spatial autocorrelation parameter (ρS)	0.40	0.02 to 0.90	490.4	1.1
Temporal autocorrelation parameter (ρT)	0.36	0.01 to 0.88	735.9	1
Variance parameter (τ^2^)	0.01	0.002 to 0.043	239.6	1.3

**Legend: β**, posterior mean; **CrI**, credible interval; **RR**, relative risk; **ESS**, effective sample size; **PSRF**, potential scale reduction factor.

## Data Availability

The data used in this study are publicly available at the following links [29,30,31,32,33,35,36].

## Supplementary Materials

The following supporting information can be downloaded at: https://www.mdpi.com/article/10.3390/v18090943/s1, Table S1: Bayesian univariable analyses of the association between each covariate and the outcome. Table S2A: Posterior regression coefficients (β), relative risks (RR), and 95% credible intervals for the primary Bayesian CAR model specifications, comprising the seven selected models fitted both with and without minimum temperature at lag 2. Table S2B: Posterior regression coefficients (β), relative risks (RR), and 95% credible intervals from the 26-selected Bayesian CAR model for sensitivity analysis. Table S3A: Covariate structure and model-comparison indices, complexity measures, and convergence diagnostics for the seven primary lag-2 CAR models (with equid cases as sentinel covariate). Table S3B: Covariate structure and model-comparison indices, complexity measures, and convergence diagnostics for the 26 sensitivity lag-2 CAR models (environmental and ecological covariates only, without equid cases). Highlighted in red the selected model. Table S4: Covariate structure, model-comparison indicesand convergence diagnostics for candidate lag-2 Bayesian CAR models with equid WNV cases as a sentinel covariate, comparing specifications with and without minimum temperature and additional environmental predictors to support selection of the final model (*CAR*_lag2_7). Highlighted in red the selected model. Figure S1: Monthly municipality-to-region IRR. Figure shows the monthly municipality-to-region model-based incidence rate ratio (IRR) for human WNV cases. Across the study period, model-estimated incidence was spatially heterogeneous, with several municipalities in northern Apulia and in the southern part of the region showing IRRs above the corresponding monthly regional average. The pattern was not restricted to the months in which human cases were observed; rather, moderately elevated IRRs were visible across much of the year, particularly in the northern cluster and in selected southern municipalities. During late summer and autumn, elevated IRRs remained evident in these areas, although most municipalities had IRRs below the regional monthly average. Figure S2: Posterior probability of elevated IRR. Figure shows the posterior probability that the municipality-to-region monthly IRR exceeded 1. Posterior support for above-average incidence was strongest and most spatially concentrated in southern municipalities during the early months of the year and again in late spring, whereas a northern cluster showed the most consistent high-probability pattern from July to November. In contrast, several municipalities with posterior median IRR above 1 had only intermediate posterior probabilities, indicating substantial uncertainty around the estimated elevation. Overall, the probability maps suggest that the apparent spatial pattern should be interpreted cautiously, particularly outside the areas with probabilities approaching 0.95 or higher.

## Abbreviations

The following abbreviations are used in this manuscript:

VBD	Vector-borne disease
WNV	West Nile virus
WND	West Nile disease
WNND	West Nile neuroinvasive disease
EWS	Early warning system
GBIF	Global Biodiversity Information Facility
CAR	Conditional Autoregressive
VIF	Variance Inflation Factor
MCMC	Markov Chain Monte Carlo
PSRF	Gelman–Rubin Potential Scale Reduction Factor
ESS	Effective Sample Size
DIC	Deviance Information Criterion
WAIC	Watanabe–Akaike Information Criterion
LMPL	Log Marginal Predictive Likelihood
pD	Effective number of parameters
pW	Effective number of parameters for WAIC
GADM	Database of Global Administrative Areas
GID_3	Geographic identifier for third-level administrative units (municipal level)
IRR	Incidence Rate Ratio
RR	Relative Risk
CrI	Credible Interval
