# Supplementary material for: Exploratory Associations Between Climatic, Environmental, and Surveillance Indicators and Human West Nile Virus Infections in Apulia: A Bayesian Spatio-Temporal Analysis
Source: Viruses. 2026 Aug 28;18(9):943. doi: 10.3390/v18090943 (PMC13611936; doi:10.3390/v18090943)

Supplementary Material

The S1 Table reports the results of the Bayesian univariable analyses. For each parameter, the posterior mean (β) with its 95% credible interval (CrI) and the corresponding relative risk (RR) with 95% CrI are shown. Variables for which the RR 95% CrI does not include 1 show a possible association with the outcome, with RR > 1 indicating an increased risk and RR < 1 indicating a inverse association. Fixed-effect coefficients generally showed PSRF values close to 1 and adequate effective sample sizes. Convergence diagnostics varied across model specifications, particularly for latent variance parameters, and are reported in the corresponding tables. The DIC, WAIC, and LMPL indices provide a summary measure of the ability of each univariable model to describe the data, suggesting that parameters associated with lower DIC/WAIC and higher LMPL values have better complexity measures. Overall, the strongest and most coherent patterns emerged for temperature-related variables, whereas land-cover and ecological indicators showed weaker and more uncertain associations.

Among static environmental variables, land-use change showed an inverse association with WNV infection risk (RR = 0.70; 95% CrI: 0.25 to 1.46), although with substantial uncertainty. Green area coverage was centered around the null value (RR = 0.94, 95% CrI: 0.84 to 1.03), indicating no clear evidence of either a protective or risk-enhancing effect. Water bodies extent showed a consistently inverse association across all lag structures examined, with RR estimates ranging from 0.997 to 0.998, however, the corresponding 95% credible intervals remained very close to the null value in all cases, indicating only a weak and stable protective tendency.

Precipitation displayed a time-dependent pattern. The contemporaneous effect was essentially near to the null value (RR = 0.99, 95% CrI: 0.95 to 1.02), while lags 1 to 3 and 6 showed inverse associations. The strongest effect was observed at lag 2, where higher precipitation was associated with lower WNV risk (RR = 0.97, 95% CrI: 0.93 to 1.0). At longer lags, the direction of the association shifted slightly towards positive values, although the estimates remained close to 1 and compatible with no clear effect.

Maximum temperature showed a positive association with WNV risk at shorter lags. The contemporaneous monthly maximum temperature was associated with increased risk (RR = 1.19, 95% CrI: 1.05 to 1.40), and the association became stronger at lag 1 (RR = 1.45, 95% CrI: 1.17 to 1.98) and peaked at lag 2 (RR = 1.59, 95% CrI: 1.22 to 2.33). A weaker but still positive association persisted at lag 3 (RR = 1.19, 95% CrI: 1.05 to 1.38), whereas estimates at lag 4 were close to the null. At longer delays, the direction reversed, with inverse associations at lag 5 (RR = 0.89, 95% CrI: 0.76 to 1.02) and especially lag 6 (RR = 0.77, 95% CrI: 0.61 to 0.93).

A very similar pattern was observed for minimum temperature. Contemporaneous minimum temperature was positively associated with WNV risk (RR = 1.20, 95% CrI: 1.0 to 1.44), with stronger associations at lag 1 (RR = 1.41, 95% CrI: 1.15 to 1.84) and lag 2 (RR = 1.49, 95% CrI: 1.18 to 2.01). The effect remained positive at lag 3 (RR = 1.20, 95% CrI: 1.04 to 1.42), approached the null at lag 4, and then reversed at longer lags, reaching an inverse association at lag 6 (RR = 0.72, 95% CrI: 0.57 to 0.88).

The avian occurrences were inversely associated with WNV risk across all lag structures examined, with RR estimates ranging from 0.29 to 0.84. The strongest inverse estimate was observed at lag 2 (RR = 0.29, 95% CrI: 0.02 to 1.09), although the corresponding interval remained wide. Overall, these results suggest a recurrent inverse tendency, but with considerable uncertainty and no stable temporal pattern.

Convergence diagnostics supported the numerical stability of the univariable models. Effective sample sizes were consistently high, and PSRF values were equal to 1.0 for all parameters, indicating satisfactory mixing and convergence of the MCMC chains.

Table S1 Bayesian univariable analyses of the association between each covariate and the outcome.

Parameter	*Posterior mean (β)*	*95% CrI*	*Relative Risk (RR)*	*95% CrI for RR*	*ESS*	*PSRF*	*DIC*	*WAIC*
Equines	3.5938	0.44 to 5.67	36.3720	1.55 to 291.43	26880.9	1	92.1936542	92.9851412
Land-use change	-0.3552	-1.39 to 0.38	0.7010	0.25 to 1.46	111867.5	1	97.8980827	97.8314709
Precipitation	-0.0125	-0.05 to 0.02	0.9876	0.95 to 1.02	155825.6	1	97.8008171	97.1407478
Precipitation (lag 1)	-0.0283	-0.07 to 0.01	0.9721	0.93 to 1.01	115578.8	1	96.0048551	95.8798483
Precipitation (lag 2)	-0.0328	-0.07 to 0	0.9677	0.93 to 1	107279.1	1	95.308208	95.2355987
Precipitation (lag 3)	-0.0155	-0.05 to 0.02	0.9846	0.95 to 1.02	144504.2	1	97.4908292	98.2840878
Precipitation (lag 4)	0.0090	-0.02 to 0.04	1.0090	0.98 to 1.04	145206.9	1	97.6770746	97.5398903
Precipitation (lag 5)	0.0089	-0.02 to 0.03	1.0089	0.98 to 1.04	144605.9	1	97.6778881	96.940316
Precipitation (lag 6)	-0.0023	-0.03 to 0.02	0.9977	0.97 to 1.03	155020.7	1	98.2006629	98.0036735
Maximum temperature	0.1766	0.05 to 0.34	1.1932	1.05 to 1.4	45837.8	1	90.6543291	90.2967163
Maximum temperature (lag 1)	0.3729	0.15 to 0.68	1.4519	1.17 to 1.98	6724.0	1	81.3186531	81.1399546
Maximum temperature (lag 2)	0.4667	0.2 to 0.85	1.5947	1.22 to 2.33	4235.9	1	78.1759829	78.0066085
Maximum temperature (lag 3)	0.1726	0.05 to 0.33	1.1884	1.05 to 1.38	48864.9	1	90.7921982	90.7584588
Maximum temperature (lag 4)	0.0192	-0.09 to 0.13	1.0194	0.91 to 1.14	157881.8	1	98.0569175	97.8484225
Maximum temperature (lag 5)	-0.1109	-0.27 to 0.02	0.8950	0.76 to 1.02	120048.1	1	95.8955467	95.614658
Maximum temperature (lag 6)	-0.2632	-0.5 to -0.08	0.7686	0.61 to 0.93	33276.4	1	89.8898411	89.6785802
Minimum temperature	0.1848	0.04 to 0.36	1.2030	1.04 to 1.44	65388.2	1	92.0476427	91.4576477

Minimum temperature (lag 1)	0.3429	0.14 to 0.61	1.4090	1.15 to 1.84	17507.2	1	84.5687962	83.9983939
Minimum temperature (lag 2)	0.3937	0.17 to 0.7	1.4825	1.18 to 2.01	11994.6	1	82.5424797	82.450341
Minimum temperature (lag 3)	0.1826	0.04 to 0.35	1.2003	1.04 to 1.42	71787.5	1	92.0738694	92.2611255
Minimum temperature (lag 4)	0.0119	-0.12 to 0.14	1.0120	0.88 to 1.15	128916.6	1	98.1884593	98.2104688
Minimum temperature (lag 5)	-0.1278	-0.29 to 0.02	0.8800	0.74 to 1.02	110303.8	1	95.5716462	95.4372797
Minimum temperature (lag 6)	-0.3280	-0.56 to -0.13	0.7204	0.57 to 0.88	26493.2	1	86.5114785	86.4146433
Avian occurrences (lag 1)	-0.1787	-0.97 to 0.17	0.8364	0.38 to 1.18	72149.8	1	97.7561779	99.4675558
Avian occurrences (lag 2)	-1.2289	-4.14 to 0.09	0.2926	0.02 to 1.09	23161.7	1	97.5104142	98.8142837
Avian occurrences (lag 3)	-0.3096	-1.37 to 0.17	0.7337	0.25 to 1.19	74232.6	1	97.8398333	98.5456982
Avian occurrences (lag 4)	-0.1754	-0.98 to 0.18	0.8391	0.37 to 1.2	70327.2	1	97.6940356	98.0580376
Avian occurrences (lag 5)	-0.3144	-1.39 to 0.17	0.7302	0.25 to 1.18	82299.9	1	97.8108602	98.0741359
Avian occurrences (lag 6)	-0.2996	-1.38 to 0.19	0.7411	0.25 to 1.21	43922.8	1	97.9154699	98.5847247
Avian occurrences	-0.1861	-0.98 to 0.16	0.8302	0.38 to 1.18	85664.1	1	97.7796761	99.4932306
Green area extent	-0.0646	-0.18 to 0.03	0.9374	0.84 to 1.03	91409.9	1	96.8998837	97.5016956
Water bodies extent	-0.0023	-0.01 to 0	0.9977	0.99 to 1	75874.3	1	97.8415577	98.7957523
Water bodies extent (lag 1)	-0.0024	-0.01 to 0	0.9976	0.99 to 1	47814.6	1	97.9504045	98.9921221
Water bodies extent (lag 2)	-0.0021	-0.01 to 0	0.9979	0.99 to 1	69254.9	1	97.888612	99.019528
Water bodies extent (lag 3)	-0.0025	-0.01 to 0	0.9975	0.99 to 1	27249.3	1	98.1669197	99.2402673
Water bodies extent (lag 4)	-0.0027	-0.01 to 0	0.9973	0.99 to 1	62239.5	1	97.8425453	99.1053385
Water bodies extent (lag 5)	-0.0023	-0.01 to 0	0.9977	0.99 to 1	89235.4	1	97.8745249	99.0524454
Water bodies extent (lag 6)	-0.0034	-0.01 to 0	0.9966	0.99 to 1	78870.7	1	97.7948332	97.9967081

Legend: ***β***, *posterior mean;* ***CrI***, *credible interval;* ***RR***, *relative risk;* ***ESS***, *effective sample size;* ***PSRF***, *potential scale reduction factor;* ***DIC***, *deviance information criterion;* ***WAIC***, *Watanabe–Akaike (widely applicable) information criterion;* ***LMPL***, *log marginal pseudo-likelihood.*

A total of 40 Bayesian spatio–temporal CAR models were fitted using the ST.CARar() function from the CARBayesST package, including 7 primary models with both maximum and minimum temperature at a 2-month lag and 7 corresponding models with only maximum temperature at a 2-month lag, plus an extended set of 26 sensitivity models comprising all admissible combinations of the selected environmental and ecological covariates at lag-2 covariates (subject to VIF < 5), fitted together with the temperature core but without equid cases. Lag–specific candidate predictors were selected on the basis of the univariable results, and biological plausibility, and the multivariable modelling strategy focused on evaluating whether predictors measured 1 month earlier or 2 months earlier improved prediction of human WNV infections incidence; because model fit and effect estimates were very similar for the 1-month and 2-month structures, the 2-month lag was retained as the reference specification.

All models assumed Poisson likelihood for the count outcome, with log-transformed municipal population (2023) included as an offset, thereby modelling relative risk while accounting for the underlying population at risk. Each model included lagged temperature variables (maximum, and in most specifications also minimum, temperature at lag 2) and alternative combinations of precipitation and environmental/ecological covariates (precipitation at lag 2, water body extent, land-use change, avian occurrences reported, green area coverage).

Spatial dependence was specified through the areal adjacency matrix (W), while temporal dependence was modelled using a first–order autoregressive structure (AR = 1) within the spatio–temporal random effects. The models were estimated in a Bayesian framework by Markov chain Monte Carlo with 3 parallel chains, 50,000 burn–in iterations, 270,000 total posterior samples, and thinning every 5 iterations.

For each model parameter, Table S2A and S2B report the posterior mean regression coefficient (β), the lower and upper bounds of the 95% credible interval (lo, hi), and the exponentiated posterior estimates expressed as relative risks (RR, with lower and upper 95% CrI) for all 40 fitted models — Table S2A for the 14 primary models (with and without minimum temperature) and Table S2B for the 26 sensitivity models excluding equid cases — while Table S3A and S3B summarize, for the seven best-performing primary models and the 26 sensitivity models respectively, the corresponding model-comparison indices, complexity measures, and convergence diagnostics. Convergence and sampling diagnostics are summarized using the effective sample size (ESS) and the potential scale reduction factor (PSRF). The significant indicator identifies parameters whose 95% credible interval excluded the null value.

Table S2A Posterior regression coefficients (β), relative risks (RR), and 95% credible intervals for the primary Bayesian CAR model specifications, comprising the seven selected models fitted both with and without minimum temperature at lag 2.

Model	*Parameter*	*Posterior mean (β)*	*95% CrI*	*Relative Risk (RR)*	*95% CrI for RR*	*ESS*	*PSRF*
CAR_lag2_1 with equids	Equids	2.37	-0.87 to 4.59	10.67	0.42 to 98.86	6469.60	1.0
	Maximum temperature (lag 2)	0.75	0.16 to 1.36	2.12	1.18 to 3.91	1807.00	1.0
	Spatial autocorrelation parameter (ϱS)	0.41	0.02 to 0.93			484.50	1.0

	Temporal autocorrelation parameter (ϱT)	0.36	0.01 to 0.92			712.00	1.1
	Precipitation (lag 2)	0.02	-0.03 to 0.08	1.02	0.97 to 1.08	20202.60	1.0
	Variance parameter (τ^2)	0.02	0 to 0.12			190.40	1.2
	Water body extent	0.00	-0.01 to 0	1.00	0.99 to 1	110578.20	1.0
CAR_lag2_1 with equids and without minimum temperature	Minimum temperature (lag 2)	-0.32	-1.02 to 0.36	0.73	0.36 to 1.43	2231.60	1.0
	Equids	2.24	-0.91 to 4.39	9.44	0.4 to 80.58	10029.10	1.0
	Maximum temperature (lag 2)	0.53	0.22 to 0.97	1.71	1.24 to 2.63	3920.30	1.0
	Spatial autocorrelation parameter (ϱS)	0.41	0.02 to 0.91			556.60	1.1
	Temporal autocorrelation parameter (ϱT)	0.36	0.01 to 0.92			673.90	1.0
	Precipitation (lag 2)	0.03	-0.02 to 0.08	1.03	0.98 to 1.08	26415.70	1.0
	Variance parameter (τ^2)	0.01	0 to 0.06			250.30	1.4
	Water body extent	0.00	-0.01 to 0	1.00	0.99 to 1	56013.30	1.0
CAR_lag2_7 with equids	Equids	3.01	-0.34 to 5.7	20.21	0.72 to 297.85	4175.70	1.0
	Maximum temperature (lag 2)	0.59	0 to 1.22	1.81	1 to 3.39	1279.30	1.0
	Spatial autocorrelation parameter (ϱS)	0.39	0.02 to 0.9			554.10	1.0
	Temporal autocorrelation parameter (ϱT)	0.34	0.01 to 0.88			843.10	1.0
	Variance parameter (τ^2)	0.03	0 to 0.14			256.40	3.1
	Water body extent	0.00	-0.01 to 0	1.00	0.99 to 1	21745.90	1.0
	Green area coverage	-0.07	-0.21 to 0.04	0.93	0.81 to 1.04	6875.50	1.0
	Minimum temperature (lag 2)	-0.23	-0.94 to 0.45	0.80	0.39 to 1.57	1576.70	1.0
CAR_lag2_7 with equids and without minimum temperature	Equids	3.04	-0.3 to 5.63	20.88	0.74 to 277.41	8252.70	1.0
	Maximum temperature (lag 2)	0.42	0.16 to 0.8	1.53	1.18 to 2.23	4863.00	1.0
	Spatial autocorrelation parameter (ϱS)	0.40	0.02 to 0.9			490.40	1.1
	Temporal autocorrelation parameter (ϱT)	0.36	0.01 to 0.88			735.90	1.0
	Variance parameter (τ^2)	0.01	0 to 0.04			239.60	1.3
	Water body extent	0.00	-0.01 to 0	1.00	0.99 to 1	54965.90	1.0
	Green area coverage	-0.09	-0.21 to 0.02	0.92	0.81 to 1.03	24792.30	1.0
CAR_lag2_m0 with equids, maximum and minimum temperature	Equids	2.25	-0.9 to 4.36	9.49	0.41 to 77.98	8162.60	1.0
	Maximum temperature (lag 2)	0.72	0.12 to 1.32	2.06	1.13 to 3.75	2292.90	1.0
	Spatial autocorrelation parameter (ϱS)	0.40	0.02 to 0.9			501.30	1.1
	Temporal autocorrelation parameter (ϱT)	0.39	0.01 to 0.95			518.60	1.0
	Variance parameter (τ^2)	0.01	0 to 0.07			270.10	1.0

	Minimum temperature (lag 2)	-0.36	-1.04 to 0.32	0.70	0.35 to 1.38	2917.20	1.0
	Equids	2.14	-1 to 4.25	8.53	0.37 to 69.9	13321.30	1.0
	Maximum temperature (lag 2)	0.45	0.18 to 0.86	1.57	1.2 to 2.35	5441.10	1.0
CAR_lag2_m0_nomin – with equids and maximum temperature only	Spatial autocorrelation parameter (ϱS)	0.39	0.02 to 0.91			551.40	1.0
	Temporal autocorrelation parameter (ϱT)	0.37	0.01 to 0.95			612.30	1.0
	Variance parameter (τ^2)	0.01	0 to 0.06			284.30	1.2
	Equids	2.37	-0.91 to 4.61	10.70	0.4 to 100.59	7708.50	1.0
	Maximum temperature (lag 2)	0.75	0.16 to 1.36	2.11	1.17 to 3.9	2184.30	1.0
	Spatial autocorrelation parameter (ϱS)	0.42	0.02 to 0.92			494.20	1.0
	Temporal autocorrelation parameter (ϱT)	0.36	0.01 to 0.92			612.70	1.0
CAR_lag2_m1 – with equids, maximum and minimum temperature, and precipitation	Precipitation (lag 2)	0.02	-0.03 to 0.08	1.02	0.97 to 1.08	25140.80	1.0
	Variance parameter (τ^2)	0.01	0 to 0.06			252.70	1.0
	Minimum temperature (lag 2)	-0.32	-1.02 to 0.37	0.73	0.36 to 1.45	2716.40	1.0
	Equids	2.30	-0.87 to 4.44	9.96	0.42 to 84.46	11559.30	1.0
	Maximum temperature (lag 2)	0.53	0.21 to 0.97	1.71	1.24 to 2.65	4427.50	1.0
	Spatial autocorrelation parameter (ϱS)	0.41	0.02 to 0.91			525.60	1.0
	Temporal autocorrelation parameter (ϱT)	0.34	0.01 to 0.87			880.10	1.0
	Precipitation (lag 2)	0.03	-0.02 to 0.08	1.03	0.98 to 1.08	29728.50	1.0
CAR_lag2_m1 with equids, maximum temperature and precipitation	Variance parameter (τ^2)	0.02	0 to 0.07			263.70	1.2
	Equids	2.58	-0.69 to 5.11	13.23	0.5 to 166.07	12268.00	1.1
	Maximum temperature (lag 2)	0.80	0.21 to 1.31	2.23	1.23 to 3.72	215.70	1.4
	Spatial autocorrelation parameter (ϱS)	0.38	0.01 to 0.92			466.00	1.0
	Temporal autocorrelation parameter (ϱT)	0.37	0.01 to 0.91			672.10	1.0
	Precipitation (lag 2)	0.03	-0.02 to 0.08	1.03	0.98 to 1.09	2116.10	1.1
	Variance parameter (τ^2)	0.02	0 to 0.1			232.60	1.3
CAR_lag2_3	Avian occurrences	-0.11	-0.85 to 0.22	0.90	0.43 to 1.25	3321.60	1.0
	Minimum temperature (lag 2)	-0.34	-0.97 to 0.4	0.71	0.38 to 1.49	286.40	1.3
	Equids	2.30	-0.85 to 4.44	10.01	0.43 to 84.55	8502.30	1.0
	Maximum temperature (lag 2)	0.55	0.22 to 1	1.73	1.24 to 2.72	3093.80	1.0
	Spatial autocorrelation parameter (ϱS)	0.39	0.02 to 0.9			570.60	1.0
	Temporal autocorrelation parameter (ϱT)	0.35	0.01 to 0.92			773.20	1.0
	Precipitation (lag 2)	0.03	-0.03 to 0.08	1.03	0.98 to 1.08	20401.90	1.0

	Variance parameter (τ²)	0.02	0 to 0.09			254.00	1.3
CAR_lag2_3 with equids and without minimum temperature	Avian occurrences	-0.12	-0.89 to 0.23	0.89	0.41 to 1.26	71902.90	1.0
	Equids	3.11	-0.27 to 5.68	22.37	0.76 to 292.22	8213.10	1.1
	Maximum temperature (lag 2)	0.66	0.03 to 1.32	1.93	1.03 to 3.74	408.70	1.0
	Spatial autocorrelation parameter (ϱS)	0.39	0.02 to 0.94			486.80	1.0
	Temporal autocorrelation parameter (ϱT)	0.35	0.01 to 0.86			851.80	1.0
	Variance parameter (τ²)	0.03	0 to 0.24			249.20	1.3
	Precipitation (lag 2)	0.02	-0.03 to 0.07	1.02	0.97 to 1.08	3885.30	1.0
CAR_lag2_4	Green area coverage	-0.06	-0.19 to 0.06	0.94	0.83 to 1.06	1992.80	1.5
	Minimum temperature (lag 2)	-0.23	-0.94 to 0.5	0.79	0.39 to 1.65	517.90	1.1
	Equids	2.95	-0.48 to 5.51	19.18	0.62 to 247.77	8563.60	1.0
	Maximum temperature (lag 2)	0.49	0.18 to 0.93	1.64	1.2 to 2.53	4332.70	1.0
	Spatial autocorrelation parameter (ϱS)	0.41	0.02 to 0.92			582.20	1.0
	Temporal autocorrelation parameter (ϱT)	0.33	0.01 to 0.84			1000.30	1.0
CAR_lag2_4 without minimum temperature	Precipitation (lag 2)	0.02	-0.03 to 0.07	1.02	0.97 to 1.08	27330.80	1.0
	Variance parameter (τ²)	0.02	0 to 0.09			311.50	1.5
	Green area coverage	-0.07	-0.2 to 0.04	0.94	0.82 to 1.04	26668.80	1.0
	Equids	2.47	-0.75 to 4.7	11.84	0.47 to 109.94	9154.30	1.0
	Maximum temperature (lag 2)	0.77	0.11 to 1.36	2.16	1.12 to 3.89	133.30	1.3
CAR_lag2_2	Spatial autocorrelation parameter (ϱS)	0.41	0.02 to 0.89			574.10	1.0
	Temporal autocorrelation parameter (ϱT)	0.34	0.01 to 0.91			747.90	1.0
	Precipitation (lag 2)	0.02	-0.02 to 0.08	1.02	0.98 to 1.08	1824.70	1.0
	Variance parameter (τ²)	0.01	0 to 0.05			263.90	1.1
	Land-use change	-0.15	-1.24 to 0.62	0.86	0.29 to 1.85	1438.50	1.0
	Minimum temperature (lag 2)	-0.32	-1.04 to 0.47	0.72	0.35 to 1.6	149.90	1.3
	Equids	2.21	-0.92 to 4.4	9.16	0.4 to 81.51	9850.40	1.0
CAR_lag2_2 without minimum temperature	Maximum temperature (lag 2)	0.54	0.22 to 0.98	1.71	1.24 to 2.66	3971.00	1.0
	Spatial autocorrelation parameter (ϱS)	0.41	0.02 to 0.91			560.40	1.1
	Temporal autocorrelation parameter (ϱT)	0.37	0.01 to 0.91			720.80	1.0
	Precipitation (lag 2)	0.03	-0.02 to 0.08	1.03	0.98 to 1.08	25899.40	1.0
	Variance parameter (τ²)	0.01	0 to 0.05			273.30	1.1
	Land-use change	-0.31	-1.43 to 0.5	0.74	0.24 to 1.65	117761.70	1.0

Legend: *β*, *posterior mean;* ***CrI****, credible interval;* ***RR****, relative risk;* ***ESS****, effective sample size;* ***PSRF****, potential scale reduction factor.*

Table S2B Posterior regression coefficients (β), relative risks (RR), and 95% credible intervals from the 26-selected Bayesian CAR model for sensitivity analysis.

Model	*Parameter*	*Posterior mean (β)*	*95% CrI*	*Relative Risk (RR)*	*95% CrI for RR*	*ESS*	*PSRF*
CAR_lag2_1	Maximum temperature (lag 2)	0.72	0.13 to 1.29	2.05	1.14 to 3.63	2070.70	1.0
	Spatial autocorrelation parameter (ϱS)	0.41	0.02 to 0.92			524.70	1.1
	Temporal autocorrelation parameter (ϱT)	0.35	0.01 to 0.91			768.00	1.1
	Variance parameter (τ^2)	0.02	-0.03 to 0.07			23685.00	1.0
	Precipitation (lag 2)	0.01	0 to 0.06	1.01	1 to 1.06	241.50	1.5
	Water body extent	0.00	-0.01 to 0	1.00	0.99 to 1	40146.70	1.0
	Minimum temperature (lag 2)	-0.27	-0.93 to 0.41	0.76	0.39 to 1.5	2580.20	1.0
CAR_lag2_2	Maximum temperature (lag 2)	0.71	0.1 to 1.34	2.03	1.1 to 3.82	1793.00	1.0
	Spatial autocorrelation parameter (ϱS)	0.40	0.02 to 0.9			528.60	1.0
	Temporal autocorrelation parameter (ϱT)	0.39	0.01 to 0.94			545.80	1.0
	Variance parameter (τ^2)	0.02	-0.03 to 0.07			23751.10	1.0
	Minimum temperature (lag 2)	0.01	0 to 0.08	1.01	1 to 1.08	248.40	1.3
	Precipitation (lag 2)	-0.23	-1.4 to 0.61	0.79	0.25 to 1.84	11690.20	1.0
	Land-use change	-0.24	-0.97 to 0.49	0.79	0.38 to 1.62	2215.10	1.0
CAR_lag2_3	Maximum temperature (lag 2)	0.72	0.15 to 1.29	2.06	1.16 to 3.64	1858.30	1.0
	Temporal autocorrelation parameter (ϱT)	0.41	0.02 to 0.92			553.20	1.0
	Spatial autocorrelation parameter (ϱS)	0.37	0.01 to 0.9			692.40	1.1
	Variance parameter (τ^2)	0.02	-0.03 to 0.07			19975.60	1.0
	Precipitation (lag 2)	0.02	0 to 0.08	1.02	1 to 1.08	304.00	1.3
	Minimum temperature (lag 2)	-0.11	-0.89 to 0.24	0.89	0.41 to 1.28	49789.60	1.0
	Avian occurrences (lag 2)	-0.27	-0.93 to 0.4	0.76	0.39 to 1.5	2253.40	1.0
CAR_lag2_4	Maximum temperature (lag 2)	0.69	0.08 to 1.33	1.99	1.08 to 3.77	1988.50	1.0
	Spatial autocorrelation parameter (ϱS)	0.37	0.01 to 0.91			478.20	1.1
	Temporal autocorrelation parameter (ϱT)	0.35	0.01 to 0.92			770.90	1.1
	Green area coverage	0.02	0 to 0.13	1.02	1 to 1.13	177.30	1.0
	Variance parameter (τ^2)	0.02	-0.03 to 0.07			26747.70	1.0

	Precipitation (lag 2)	-0.02	-0.14 to 0.07	0.98	0.87 to 1.08	11771.20	1.0
	Minimum temperature (lag 2)	-0.25	-0.98 to 0.46	0.78	0.38 to 1.58	2424.10	1.0
CAR_lag2_5	Maximum temperature (lag 2)	0.70	0.08 to 1.35	2.02	1.08 to 3.84	1560.20	1.0
	Spatial autocorrelation parameter (ϱS)	0.40	0.02 to 0.92			490.60	1.0
	Temporal autocorrelation parameter (ϱT)	0.37	0.01 to 0.91			668.90	1.0
	Variance parameter (τ^2)	0.04	0 to 0.4			206.40	4.2
	Water body extent	0.00	-0.01 to 0	1.00	0.99 to 1	36986.90	1.0
	Minimum temperature (lag 2)	-0.17	-1.35 to 0.68	0.85	0.26 to 1.98	11267.90	1.0
	Land-use change	-0.31	-1.04 to 0.41	0.73	0.35 to 1.5	1935.80	1.0
CAR_lag2_6	Maximum temperature (lag 2)	0.72	0.13 to 1.32	2.05	1.14 to 3.74	1544.60	1.0
	Spatial autocorrelation parameter (ϱS)	0.40	0.02 to 0.92			530.40	1.1
	Temporal autocorrelation parameter (ϱT)	0.35	0.01 to 0.88			770.30	1.0
	Variance parameter (τ^2)	0.02	0 to 0.08			180.00	1.3
	Water body extent	0.00	-0.01 to 0	1.00	0.99 to 1	33265.30	1.0
	Minimum temperature (lag 2)	-0.12	-0.89 to 0.24	0.88	0.41 to 1.27	36015.60	1.0
	Avian occurrences (lag 2)	-0.33	-1 to 0.33	0.72	0.37 to 1.39	2034.50	1.0
CAR_lag2_7	Maximum temperature (lag 2)	0.63	0.04 to 1.27	1.88	1.04 to 3.56	1912.20	1.0
	Spatial autocorrelation parameter (ϱS)	0.40	0.02 to 0.9			503.40	1.0
	Temporal autocorrelation parameter (ϱT)	0.35	0.01 to 0.88			818.90	1.0
	Variance parameter (τ^2)	0.01	0 to 0.07			188.90	1.5
	Green area coverage	0.00	-0.01 to 0	1.00	0.99 to 1	38811.20	1.0
	Water body extent	-0.03	-0.15 to 0.07	0.97	0.86 to 1.07	10914.60	1.0
	Minimum temperature (lag 2)	-0.25	-0.96 to 0.42	0.78	0.38 to 1.52	2343.00	1.0
CAR_lag2_8	Maximum temperature (lag 2)	0.71	0.08 to 1.35	2.03	1.09 to 3.88	1384.50	1.0
	Spatial autocorrelation parameter (ϱS)	0.40	0.02 to 0.94			569.70	1.1
	Temporal autocorrelation parameter (ϱT)	0.39	0.01 to 0.91			476.80	1.0
	Variance parameter (τ^2)	0.02	0 to 0.08			169.00	1.1
	Minimum temperature (lag 2)	-0.14	-0.93 to 0.24	0.87	0.4 to 1.27	49011.80	1.0
	Land-use change	-0.18	-1.35 to 0.66	0.83	0.26 to 1.93	9864.40	1.0
	Avian occurrences (lag 2)	-0.32	-1.07 to 0.41	0.73	0.34 to 1.51	1699.60	1.0
CAR_lag2_9	Maximum temperature (lag 2)	0.63	-0.03 to 1.35	1.87	0.97 to 3.85	1475.30	1.0

	Spatial autocorrelation parameter (ϱS)	0.42	0.02 to 0.94			457.80	1.0
	Temporal autocorrelation parameter (ϱT)	0.34	0.01 to 0.88			820.00	1.0
	Variance parameter (τ^2)	0.02	0 to 0.13			191.30	1.2
	Green area coverage	-0.03	-0.15 to 0.07	0.97	0.86 to 1.07	9177.50	1.0
	Minimum temperature (lag 2)	-0.23	-1.04 to 0.52	0.79	0.35 to 1.68	1804.40	1.0
	Land-use change	-0.25	-1.46 to 0.62	0.78	0.23 to 1.86	8325.60	1.0
CAR_lag2_10	Maximum temperature (lag 2)	0.68	0.08 to 1.34	1.97	1.08 to 3.81	1507.50	1.0
	Spatial autocorrelation parameter (ϱS)	0.42	0.02 to 0.94			424.40	1.0
	Temporal autocorrelation parameter (ϱT)	0.34	0.01 to 0.84			910.00	1.0
	Green area coverage	0.02	0 to 0.08	1.02	1 to 1.08	198.50	2.1
	Variance parameter (τ^2)	-0.03	-0.15 to 0.07			10407.80	1.0
	Minimum temperature (lag 2)	-0.12	-0.89 to 0.25	0.89	0.41 to 1.28	57630.80	1.0
	Avian occurrences (lag 2)	-0.29	-1.02 to 0.39	0.75	0.36 to 1.47	1886.40	1.0
CAR_lag2_11	Maximum temperature (lag 2)	0.69	0.1 to 1.33	2.00	1.1 to 3.77	1647.30	1.0
	Spatial autocorrelation parameter (ϱS)	0.41	0.02 to 0.91			551.50	1.0
	Temporal autocorrelation parameter (ϱT)	0.37	0.01 to 0.94			615.80	1.0
	Variance parameter (τ^2)	0.02	-0.03 to 0.07			21982.60	1.0
	Precipitation (lag 2)	0.02	0 to 0.1	1.02	1 to 1.1	175.50	1.5
	Minimum temperature (lag 2)	0.00	-0.01 to 0	1.00	0.99 to 1	36986.10	1.0
	Water body extent	-0.20	-1.39 to 0.65	0.82	0.25 to 1.92	11125.00	1.0
	Land-use change	-0.23	-0.96 to 0.49	0.80	0.38 to 1.63	1995.30	1.0
CAR_lag2_12	Maximum temperature (lag 2)	0.74	0.17 to 1.32	2.10	1.18 to 3.73	1829.70	1.0
	Spatial autocorrelation parameter (ϱS)	0.39	0.01 to 0.9			498.40	1.0
	Temporal autocorrelation parameter (ϱT)	0.35	0.01 to 0.9			723.50	1.0
	Variance parameter (τ^2)	0.02	-0.03 to 0.07			19389.70	1.0
	Precipitation (lag 2)	0.02	0 to 0.08	1.02	1 to 1.08	217.00	1.5
	Water body extent	0.00	-0.01 to 0	1.00	0.99 to 1	32823.60	1.0
	Minimum temperature (lag 2)	-0.11	-0.87 to 0.24	0.89	0.42 to 1.27	96268.90	1.0
	Avian occurrences (lag 2)	-0.29	-0.97 to 0.38	0.75	0.38 to 1.46	2322.20	1.0
CAR_lag2_13	Maximum temperature (lag 2)	0.66	0.06 to 1.31	1.94	1.07 to 3.71	1247.40	1.0
	Spatial autocorrelation parameter (ϱS)	0.40	0.02 to 0.9			556.70	1.0

	Temporal autocorrelation parameter (ϱT)	0.36	0.01 to 0.87			796.70	1.1
	Green area coverage	0.02	-0.03 to 0.07	1.02	0.97 to 1.07	16125.60	1.0
	Variance parameter (τ^2)	0.02	0 to 0.08			241.20	1.2
	Precipitation (lag 2)	0.00	-0.01 to 0	1.00	0.99 to 1	24783.80	1.0
	Water body extent	-0.03	-0.15 to 0.07	0.97	0.86 to 1.08	7401.40	1.0
	Minimum temperature (lag 2)	-0.21	-0.96 to 0.5	0.81	0.38 to 1.65	1427.90	1.0
CAR_lag2_14	Maximum temperature (lag 2)	0.72	0.09 to 1.39	2.05	1.1 to 4.01	1505.30	1.0
	Spatial autocorrelation parameter (ϱS)	0.39	0.02 to 0.91			535.90	1.1
	Temporal autocorrelation parameter (ϱT)	0.37	0.01 to 0.92			646.50	1.0
	Minimum temperature (lag 2)	0.02	-0.03 to 0.07	1.02	0.97 to 1.07	22685.80	1.0
	Variance parameter (τ^2)	0.02	0 to 0.08			204.80	1.2
	Precipitation (lag 2)	-0.12	-0.9 to 0.24	0.89	0.41 to 1.27	92532.20	1.0
	Land-use change	-0.20	-1.38 to 0.65	0.82	0.25 to 1.91	9450.00	1.0
	Avian occurrences (lag 2)	-0.25	-1.01 to 0.48	0.78	0.36 to 1.62	1907.30	1.0
CAR_lag2_15	Maximum temperature (lag 2)	0.66	-0.02 to 1.4	1.94	0.98 to 4.06	842.30	1.0
	Spatial autocorrelation parameter (ϱS)	0.37	0.01 to 0.91			795.80	1.1
	Temporal autocorrelation parameter (ϱT)	0.37	0.01 to 0.89			531.30	1.0
	Green area coverage	0.02	-0.03 to 0.07	1.02	0.97 to 1.07	14086.70	1.0
	Variance parameter (τ^2)	0.02	0 to 0.09			256.20	1.2
	Precipitation (lag 2)	-0.02	-0.14 to 0.08	0.98	0.87 to 1.08	5727.70	1.0
	Minimum temperature (lag 2)	-0.19	-1.04 to 0.61	0.82	0.35 to 1.84	987.60	1.0
	Land-use change	-0.25	-1.46 to 0.63	0.78	0.23 to 1.87	5901.80	1.0
CAR_lag2_16	Maximum temperature (lag 2)	0.69	0.08 to 1.36	2.00	1.09 to 3.9	901.40	1.0
	Spatial autocorrelation parameter (ϱS)	0.39	0.02 to 0.91			497.50	1.0
	Temporal autocorrelation parameter (ϱT)	0.37	0.01 to 0.94			575.60	1.1
	Green area coverage	0.02	-0.03 to 0.07	1.02	0.97 to 1.07	13175.60	1.0
	Variance parameter (τ^2)	0.02	0 to 0.09			206.10	1.1
	Precipitation (lag 2)	-0.02	-0.14 to 0.08	0.98	0.87 to 1.08	6810.10	1.0
	Minimum temperature (lag 2)	-0.11	-0.88 to 0.24	0.89	0.41 to 1.27	24552.40	1.0
	Avian occurrences (lag 2)	-0.25	-0.98 to 0.45	0.78	0.38 to 1.57	1138.20	1.0
CAR_lag2_17	Maximum temperature (lag 2)	0.73	0.12 to 1.35	2.07	1.12 to 3.84	1435.30	1.0

	Spatial autocorrelation parameter (ϱS)	0.42	0.02 to 0.91			571.40	1.1
	Temporal autocorrelation parameter (ϱT)	0.35	0.01 to 0.9			763.90	1.1
	Variance parameter (τ^2)	0.01	0 to 0.05			309.60	1.0
	Water body extent	0.00	-0.01 to 0	1.00	0.99 to 1	31644.00	1.0
	Minimum temperature (lag 2)	-0.13	-1.3 to 0.71	0.88	0.27 to 2.03	9308.30	1.0
	Land-use change	-0.13	-0.92 to 0.24	0.88	0.4 to 1.27	97541.10	1.0
	Avian occurrences (lag 2)	-0.34	-1.07 to 0.37	0.71	0.34 to 1.45	1778.60	1.0
CAR_lag2_18	Maximum temperature (lag 2)	0.60	-0.04 to 1.27	1.83	0.96 to 3.56	780.20	1.0
	Spatial autocorrelation parameter (ϱS)	0.41	0.02 to 0.9			578.80	1.0
	Temporal autocorrelation parameter (ϱT)	0.35	0.01 to 0.86			754.20	1.0
	Variance parameter (τ^2)	0.01	0 to 0.06			267.00	1.2
	Green area coverage	0.00	-0.01 to 0	1.00	0.99 to 1	16982.20	1.0
	Water body extent	-0.04	-0.16 to 0.06	0.97	0.85 to 1.07	6369.50	1.0
	Minimum temperature (lag 2)	-0.21	-1.41 to 0.66	0.81	0.24 to 1.93	6783.90	1.0
	Land-use change	-0.21	-0.97 to 0.53	0.81	0.38 to 1.7	918.30	1.0
CAR_lag2_19	Maximum temperature (lag 2)	0.64	0.04 to 1.29	1.90	1.04 to 3.63	855.60	1.0
	Spatial autocorrelation parameter (ϱS)	0.43	0.02 to 0.92			550.40	1.1
	Temporal autocorrelation parameter (ϱT)	0.37	0.01 to 0.93			698.30	1.2
	Green area coverage	0.01	0 to 0.05	1.01	1 to 1.05	305.50	1.1
	Variance parameter (τ^2)	0.00	-0.01 to 0			17369.40	1.0
	Water body extent	-0.03	-0.16 to 0.07	0.97	0.85 to 1.07	6188.30	1.0
	Minimum temperature (lag 2)	-0.11	-0.87 to 0.24	0.89	0.42 to 1.28	20892.40	1.0
	Avian occurrences (lag 2)	-0.26	-0.97 to 0.44	0.77	0.38 to 1.56	1064.10	1.0
CAR_lag2_20	Maximum temperature (lag 2)	0.65	-0.04 to 1.33	1.91	0.96 to 3.8	588.90	1.1
	Spatial autocorrelation parameter (ϱS)	0.39	0.01 to 0.92			535.90	1.0
	Temporal autocorrelation parameter (ϱT)	0.35	0.01 to 0.88			841.00	1.1
	Green area coverage	0.02	0 to 0.11	1.02	1 to 1.12	201.80	1.0
	Variance parameter (τ^2)	-0.03	-0.15 to 0.07			5803.40	1.0
	Minimum temperature (lag 2)	-0.11	-0.91 to 0.28	0.90	0.4 to 1.32	18977.70	1.1
	Land-use change	-0.20	-1.39 to 0.65	0.82	0.25 to 1.92	5000.70	1.0
	Avian occurrences (lag 2)	-0.25	-1.02 to 0.53	0.78	0.36 to 1.69	714.50	1.1

CAR_lag2_21	Maximum temperature (lag 2)	0.74	0.12 to 1.39	2.09	1.13 to 4.03	1639.00	1.0
	Spatial autocorrelation parameter (ϱS)	0.41	0.02 to 0.93			475.90	1.1
	Temporal autocorrelation parameter (ϱT)	0.36	0.01 to 0.9			653.20	1.1
	Minimum temperature (lag 2)	0.02	-0.03 to 0.07	1.02	0.97 to 1.07	20691.90	1.0
	Variance parameter (τ^2)	0.02	0 to 0.06			241.60	1.1
	Precipitation (lag 2)	0.00	-0.01 to 0	1.00	0.99 to 1	37234.50	1.0
	Water body extent	-0.12	-0.89 to 0.24	0.89	0.41 to 1.27	85496.00	1.0
	Land-use change	-0.15	-1.33 to 0.71	0.86	0.26 to 2.02	10327.60	1.0
	Avian occurrences (lag 2)	-0.27	-1.04 to 0.47	0.76	0.35 to 1.6	2035.70	1.0
CAR_lag2_22	Maximum temperature (lag 2)	0.64	-0.04 to 1.36	1.89	0.97 to 3.9	1325.80	1.0
	Spatial autocorrelation parameter (ϱS)	0.40	0.02 to 0.9			524.70	1.0
	Temporal autocorrelation parameter (ϱT)	0.37	0.01 to 0.9			720.50	1.1
	Precipitation (lag 2)	0.02	0 to 0.15	1.02	1 to 1.16	166.20	1.3
	Green area coverage	0.02	-0.03 to 0.07	1.02	0.97 to 1.07	20327.20	1.0
	Variance parameter (τ^2)	0.00	-0.01 to 0			34923.20	1.0
	Water body extent	-0.03	-0.15 to 0.07	0.97	0.86 to 1.08	7400.00	1.0
	Minimum temperature (lag 2)	-0.18	-1 to 0.61	0.84	0.37 to 1.85	1518.60	1.0
	Land-use change	-0.22	-1.45 to 0.67	0.81	0.24 to 1.95	7341.50	1.0
CAR_lag2_23	Maximum temperature (lag 2)	0.68	0.05 to 1.36	1.98	1.05 to 3.89	1553.20	1.0
	Spatial autocorrelation parameter (ϱS)	0.40	0.02 to 0.91			527.70	1.0
	Temporal autocorrelation parameter (ϱT)	0.37	0.01 to 0.93			698.60	1.0
	Green area coverage	0.02	0 to 0.15	1.02	1 to 1.16	225.60	2.4
	Variance parameter (τ^2)	0.02	-0.03 to 0.07			22464.60	1.0
	Precipitation (lag 2)	0.00	-0.01 to 0	1.00	0.99 to 1	35615.30	1.0
	Water body extent	-0.03	-0.15 to 0.07	0.97	0.86 to 1.08	9375.10	1.0
	Minimum temperature (lag 2)	-0.10	-0.87 to 0.25	0.90	0.42 to 1.28	109391.80	1.0
	Avian occurrences (lag 2)	-0.23	-0.98 to 0.49	0.80	0.38 to 1.63	1966.00	1.0
CAR_lag2_24	Maximum temperature (lag 2)	0.66	-0.01 to 1.39	1.93	0.99 to 4	1353.40	1.0
	Spatial autocorrelation parameter (ϱS)	0.38	0.01 to 0.89			566.80	1.0
	Temporal autocorrelation parameter (ϱT)	0.35	0.01 to 0.88			733.40	1.0
	Green area coverage	0.02	-0.03 to 0.07	1.02	0.97 to 1.07	22937.70	1.0

	Variance parameter (τ^2)	0.02	0 to 0.07			263.50	1.0
	Precipitation (lag 2)	-0.03	-0.15 to 0.08	0.97	0.86 to 1.08	7955.10	1.0
	Minimum temperature (lag 2)	-0.11	-0.9 to 0.25	0.89	0.41 to 1.28	112974.70	1.0
	Land-use change	-0.18	-1 to 0.6	0.83	0.37 to 1.82	1500.60	1.0
	Avian occurrences (lag 2)	-0.23	-1.45 to 0.64	0.79	0.24 to 1.9	7065.70	1.0
CAR_lag2_25	Maximum temperature (lag 2)	0.65	-0.02 to 1.41	1.91	0.98 to 4.08	1119.90	1.0
	Spatial autocorrelation parameter (ϱS)	0.41	0.02 to 0.91			538.30	1.1
	Temporal autocorrelation parameter (ϱT)	0.37	0.01 to 0.95			630.60	1.1
	Variance parameter (τ^2)	0.01	0 to 0.06			225.90	1.1
	Green area coverage	0.00	-0.01 to 0	1.00	0.99 to 1	31356.90	1.0
	Water body extent	-0.04	-0.16 to 0.07	0.97	0.85 to 1.07	6901.90	1.0
	Minimum temperature (lag 2)	-0.12	-0.9 to 0.25	0.89	0.41 to 1.28	121721.60	1.0
	Land-use change	-0.17	-1.38 to 0.71	0.85	0.25 to 2.04	7285.60	1.0
	Avian occurrences (lag 2)	-0.24	-1.07 to 0.52	0.79	0.34 to 1.69	1385.50	1.0
CAR_lag2_26	Maximum temperature (lag 2)	0.64	-0.01 to 1.34	1.90	0.99 to 3.84	1542.60	1.0
	Spatial autocorrelation parameter (ϱS)	0.41	0.02 to 0.91			564.30	1.1
	Temporal autocorrelation parameter (ϱT)	0.36	0.01 to 0.92			786.00	1.1
	Variance parameter (τ^2)	0.02	-0.03 to 0.07			22767.70	1.0
	Green area coverage	0.01	0 to 0.06	1.01	1 to 1.06	263.10	1.5
	Precipitation (lag 2)	0.00	-0.01 to 0	1.00	0.99 to 1	38809.50	1.0
	Water body extent	-0.03	-0.16 to 0.07	0.97	0.85 to 1.07	8708.70	1.0
	Minimum temperature (lag 2)	-0.11	-0.88 to 0.25	0.89	0.41 to 1.28	96498.20	1.0
	Land-use change	-0.18	-0.97 to 0.58	0.84	0.38 to 1.79	1845.60	1.0
	Avian occurrences (lag 2)	-0.19	-1.4 to 0.68	0.83	0.25 to 1.98	8896.70	1.0

Legend: **β**, posterior mean; **CrI**, credible interval; **RR**, relative risk; **ESS**, effective sample size; **PSRF**, potential scale reduction factor.

Table S3A reports the seven best–performing lag–2 Conditional Autoregressive (CAR) models identified during model selection, all including equid WNV cases as a fixed sentinel covariate together with the lag-2 temperature core (maximum temperature, and in most specifications also minimum temperature). These seven models were selected by ranking all candidate lag-2 CAR models with equids according to DIC and WAIC and then retaining the top performers while ensuring variation in covariate structure. In particular, we included the two baseline models m0 and m1 (with their counterparts without minimum temperature) because they represent simpler, highly parsimonious specifications with distinctive temperature and precipitation structures, and the lag2_7 variants with and without minimum temperature to explicitly assess the impact of excluding the minimum temperature from one of the best-fitting models.

For each model, Table S3A indicates the included environmental covariates are indicated together with measures of model-comparison indices, complexity measures, and convergence diagnostics. Model fit was assessed using the Deviance Information Criterion (DIC), while the Watanabe–Akaike Information Criterion (WAIC) and the Log Marginal Predictive Likelihood (LMPL) were considered as complementary model-comparison criteria. Lower DIC and WAIC values, and higher (less negative) LMPL values, indicate better model performance. Models with markedly higher pD or pW relative to the nominal number of fixed-effect parameters were interpreted as showing greater effective model complexity, which was considered alongside DIC/WAIC and parsimony in the final model choice, rather than applying a fixed complexity threshold. Convergence of the Markov Chain Monte Carlo (MCMC) simulations was evaluated using the Gelman–Rubin potential scale reduction factor ($\hat{R}$) for regression coefficients (β), spatial and temporal autocorrelation parameters (ϱ), and variance parameters (τ^2). Values of $\hat{R}$ close to 1 indicate satisfactory convergence across chains. Sampling efficiency was assessed using the Effective Sample Size (ESS), reported as both the minimum and mean ESS across model parameters.

The seven models presented in Table S3A showed highly comparable DIC, WAIC, and LMPL values, suggesting similar explanatory and predictive performance among specifications combining the temperature core, equid cases, and a single additional environmental covariate. Therefore, model selection among these seven specifications also considered convergence diagnostics, parameter stability, and model parsimony, in addition to fit and complexity measures.

Table S3A Covariate structure and model-comparison indices, complexity measures, and convergence diagnostics for the seven primary lag-2 CAR models (with equid cases as sentinel covariate).

**Model**	***Minimum temperature (lag 2)***	***Precipitation at lag2***	***Green areas extent***	Water bodies extent	Land-use change	Avian occurrences	DIC	pD	WAIC	pW	LMPL	Max $\hat{R}$ (β)	Max $\hat{R}$ (ϱ)	Max $\hat{R}$ (τ^2)	Minimum ESS	**Mean ESS**
CAR_lag2_m0 with equids and maximum temperature only							76.96	2.73	77.73	2.92	-39.38	1	1.01	1.12	5298.6	8020.3

CAR_lag2_m0 with equids, maximum and minimum temperature	✓						77.47	3.54	78.92	4.22	-40.27	1	1.02	1.01	2292.9	4338.5
CAR_lag2_m1 with equids, maximum temperature and precipitation		✓					77.75	3.53	78.93	3.93	-40.36	1	1.01	1.08	4427.5	12556.2
CAR_lag2_4 without minimum temperature		✓	✓				78.43	4.3	80.13	4.97	-41.19	1	1.01	1.21	4332.7	14272.0
CAR_lag2_m1 with equids, maximum and minimum temperature, and precipitation	✓	✓					78.57	4.33	80.61	5.37	-41.17	1	1.01	1.00	2184.3	8285.9
CAR_lag2_7 with equids and without minimum temperature		✓		✓			78.7	3.96	80.78	4.84	-41.77	1	1.02	1.15	4804.8	19535.7
CAR_lag2_2	✓	✓			✓		78.91	4.16	80.84	5.12	-41.68	1.1	1.01	1.07	133.3	2175.2
CAR_lag2_3 with equids and without minimum temperature		✓				✓	79.27	3.92	81.8	5.2	-42.51	1	1.01	1.13	3054.5	21391.1
CAR_lag2_1 with equids and without minimum temperature			✓	✓			79.33	3.89	81.33	4.92	-41.45	1	1.03	1.19	3920.3	20082.3
CAR_lag2_2 without minimum temperature		✓			✓		79.44	4.25	80.63	4.57	-40.9	1	1.03	1.06	3970.9	32304.3
CAR_lag2_4	✓	✓	✓				79.55	4.98	82.04	6.31	-42.19	1.16	1.01	1.15	408.7	2624.3
CAR_lag2_7 with equids	✓			✓			79.89	4.76	82.27	5.93	-42.35	1	1.01	1.35	1279.3	6359.8
CAR_lag2_3	✓	✓				✓	79.92	4.39	84.32	7.13	-43.71	1.14	1.01	1.15	215.7	3104.6
CAR_lag2_1 with equids	✓	✓		✓			80.06	4.67	82.87	6.22	-42.52	1	1.02	1.09	1807.1	24059.5

Legend: DIC = Deviance Information Criterion; **WAIC** = the Watanabe–Akaike Information Criterion; **LMPL** = Log Marginal Predictive Likelihood; **pD** = effective number of parameters; **pW** = effective number of parameters for WAIC; $\hat{\mathbf{R}}$ = Gelman–Rubin potential scale reduction factor; **ESS** = effective sample size.

Table S3B reports the extended set of 26 sensitivity Conditional Autoregressive (CAR) models fitted at lag 2, comprising all admissible combinations (VIF < 5) of the environmental and ecological covariates — precipitation, water body extent, land-use change, green area coverage, and avian occurrences reported — together with the lag-2 temperature core, but excluding equid WNV cases. This sensitivity analysis was designed to evaluate whether any environmental or ecological predictor showed an independent association with the outcome once the dominant explanatory contribution of the equid sentinel covariate was removed from the model. From this larger set, the seven best-performing sensitivity models were identified by ranking according to DIC and WAIC; priority was given to specifications including precipitation at lag 2 (preclag2), together with the

lag2_7 model without minimum temperature, which remained among the top-ranked models and was used to further evaluate the impact of removing tmin from the temperature core.

As in Table S3A, for each model the included covariates are indicated together with measures of model fit (DIC), complexity measures (WAIC, LMPL), model complexity (pD, pW), and MCMC convergence diagnostics ($\hat{R}$ for β, ρ, and τ^2; minimum and mean ESS). Lower DIC and WAIC values, higher (less negative) LMPL values, and $\hat{R}$ close to 1 indicate better model performance and satisfactory convergence, respectively.

Comparison across the 26 models in Table S3B allows the relative contribution of individual environmental and ecological covariates to be assessed in isolation from after excluding the equid sentinel covariate, thereby complementing the primary model set in Table S3A and providing a more complete picture of the observed associations.

Table S3B Covariate structure and model-comparison indices, complexity measures, and convergence diagnostics for the 26 sensitivity lag-2 CAR models (environmental and ecological covariates only, without equid cases). Highlighted in red the selected model.

Model	Precipitation at lag 2	Green areas extent	Water bodies extent	Land-use change	Avian occurrences	DIC	pD	WAIC	pW	LMPL	Max $\hat{R}$ (β)	Max $\hat{R}$ (ϱ)	$\hat{R}$ (τ^2)	Minimum ESS	Mean ESS
CAR_lag2_3	✓				✓	81.61	3.90	83.68	5.07	-42.54	1.00	1.02	1.14	1858.3	15362.9
CAR_lag2_1	✓		✓			81.82	3.94	83.45	4.83	-42.23	1.00	1.04	1.20	2070.7	14423.1
CAR_lag2_4	✓	✓				81.83	4.34	83.19	5.07	-41.79	1.00	1.02	1.01	1988.5	9404.6
CAR_lag2_2	✓			✓		81.99	4.24	82.55	4.30	-41.39	1.00	1.01	1.14	1793.0	8545.4
CAR_lag2_6			✓		✓	82.05	3.60	84.60	5.09	-43.50	1.01	1.03	1.19	1544.6	15067.7
CAR_lag2_10		✓			✓	82.07	3.92	84.65	5.46	-43.30	1.01	1.01	1.29	1507.5	14780.9
CAR_lag2_7		✓	✓			82.08	3.92	83.93	4.97	-42.37	1.00	1.01	1.18	1912.2	11484.6
CAR_lag2_9		✓		✓		82.25	4.24	83.01	4.43	-41.67	1.01	1.01	1.14	1475.3	4756.8
CAR_lag2_5			✓	✓		82.29	3.98	83.31	4.42	-42.07	1.00	1.02	1.41	1560.2	10917.5
CAR_lag2_8				✓	✓	82.32	3.92	84.05	4.74	-43.39	1.00	1.02	1.05	1384.5	12896.8
CAR_lag2_12	✓		✓		✓	83.25	4.31	86.18	6.05	-44.86	1.00	1.00	1.23	1829.7	25970.4
CAR_lag2_16	✓	✓			✓	83.29	4.65	86.08	6.30	-44.00	1.00	1.02	1.07	901.4	8020.2
CAR_lag2_11	✓		✓	✓		83.49	4.59	84.93	5.25	-42.88	1.00	1.01	1.17	1647.3	12804.8
CAR_lag2_13	✓	✓	✓			83.49	4.69	85.83	6.04	-43.43	1.00	1.03	1.17	1247.4	8912.7
CAR_lag2_19		✓	✓		✓	83.49	4.28	86.72	6.21	-44.43	1.00	1.06	1.05	855.6	8000.2
CAR_lag2_14	✓			✓	✓	83.56	4.65	85.58	5.63	-45.38	1.01	1.02	1.10	1505.3	21793.2

**CAR_lag2_18**		✓	✓	✓		83.70	4.54	85.33	5.29	-43.21	1.01	1.00	1.12	780.2	5581.4
**CAR_lag2_15**	✓	✓		✓		83.70	5.00	85.00	5.52	-42.74	1.01	1.02	1.13	842.3	4911.5
**CAR_lag2_20**		✓		✓	✓	83.74	4.68	86.02	5.81	-44.76	1.04	1.02	1.02	588.9	5397.8
**CAR_lag2_17**			✓	✓	✓	83.79	4.22	86.30	5.56	-44.41	1.00	1.03	1.03	1435.3	24066.5
**CAR_lag2_23**	✓	✓	✓		✓	84.87	5.07	88.60	7.31	-45.82	1.01	1.01	1.30	1553.2	26184.8
**CAR_lag2_24**	✓	✓		✓	✓	85.02	5.28	87.77	6.75	-44.96	1.00	1.01	1.02	1353.4	22344.6
**CAR_lag2_21**	✓		✓	✓	✓	85.17	5.03	88.20	6.70	-45.27	1.01	1.02	1.05	1639.0	22900.8
**CAR_lag2_22**	✓	✓	✓	✓		85.20	5.42	87.25	6.41	-44.14	1.00	1.02	1.12	1325.8	10807.9
**CAR_lag2_25**		✓	✓	✓	✓	85.47	5.02	88.81	6.90	-45.79	1.01	1.02	1.07	1119.9	24553.6
**CAR_lag2_26**	✓	✓	✓	✓	✓	86.42	5.61	89.91	7.55	-46.41	1.00	1.03	1.20	1542.6	22759.5

Legend: DIC = Deviance Information Criterion; WAIC = the Watanabe–Akaike Information Criterion; LMPL = Log Marginal Predictive Likelihood; pD = effective number of parameters; pW = effective number of parameters for WAIC; $\hat{R}$ = Gelman–Rubin potential scale reduction factor; ESS = effective sample size.

Table S4 summarizes the fit, complexity, and convergence diagnostics for the main lag-2 CAR models evaluated. The simplest specification (equid WNV cases and maximum temperature at lag 2 only) achieved the lowest DIC and WAIC but lacked environmental covariates for ecological interpretation. Models including minimum temperature and multiple additional predictors showed worse fit, higher effective numbers of parameters, and, in some cases, lower ESS, reflecting collinearity and over-parameterization under sparse data. Finally, parsimony and stability, as reflected by lower effective numbers of parameters (pD, pW) and higher minimum ESS, favor both CAR_lag2_m0 with equids and maximum temperature only and CAR_lag2_7 with equids and without minimum temperature with the latter retained as the selected exploratory specification because it preserved the planned environmental covariates and showed competitive fit. However, the latent variance parameter showed slower mixing, and all inferences from this specification should be interpreted cautiously.

Table S4 Covariate structure, model-comparison indicesand convergence diagnostics for candidate lag-2 Bayesian CAR models with equid WNV cases as a sentinel covariate, comparing specifications with and without minimum temperature and additional environmental predictors to support selection of the final model (*CAR*_lag2_7). Highlighted in red the selected model.

Model	***DIC***	***pD***	***WAIC***	***pW***	***LMPL***	***Max $\hat{R}$ (β)***	***Max $\hat{R}$ (ρ)***	***$\hat{R}$ (τ^2)***	***Minimum ESS***	***Mean ESS***
CAR_lag2_m0 with equids and maximum temperature only	76.96	2.73	77.73	2.92	-39.38	1	1.01	1.12	5298.59	8020.32
CAR_lag2_m0 with equids, maximum and minimum temperature	77.47	3.54	78.92	4.22	-40.27	1	1.02	1.01	2292.89	4338.50
CAR_lag2_m1 with equids, maximum temperature and precipitation	77.75	3.53	78.93	3.93	-40.36	1	1.01	1.08	4427.54	12556.19
CAR_lag2_4 without minimum temperature	78.43	4.3	80.13	4.97	-41.19	1	1.01	1.21	4332.72	14272.00
CAR_lag2_m1 with equids, maximum and minimum temperature, and precipitation	78.57	4.33	80.61	5.37	-41.17	1	1.01	1.00	2184.35	8285.90
CAR_lag2_7 with equids and without minimum temperature	78.7	3.96	80.78	4.84	-41.77	1	1.02	1.15	4804.83	19535.73
CAR_lag2_2	78.91	4.16	80.84	5.12	-41.68	1.1	1.01	1.07	133.31	2175.15

Monthly municipality-to-region model-based incidence rate ratio. To characterize the spatial distribution of model-estimated WNV risk within each month, a municipality-to-region monthly incidence rate ratio (IRR) was calculated from the posterior fitted case counts of the final Bayesian spatiotemporal model. For municipality i and month t, the municipal fitted incidence was defined as $\hat{Y}_{it}/N_i$, where $\hat{Y}_{it}$ is the model-fitted number of human WNV cases and N_i is the resident population of municipality i. The corresponding regional fitted incidence for month t was calculated as $\sum_i \hat{Y}_{it}/N_R$, where N_R is the total population of Apulia. The monthly IRR was then computed as:

$$IRR_{it\_monthly} = \frac{\hat{Y}_{it}/N_i}{\left(\sum_i \hat{Y}_{it}\right)/N_R}.$$

Thus, an IRR above 1 indicates that the model-estimated incidence in a municipality was higher than the fitted regional average in the same month, whereas values below 1 indicate lower-than-average incidence. This measure is not a standardized incidence ratio, as it does not compare observed and expected counts derived from external reference rates; rather, it is a within-month, municipality-to-region model-based incidence rate ratio. Standardized incidence ratios are conventionally defined as observed-to-expected case ratios.

When posterior samples of fitted values were available, the IRR was calculated separately for each Markov chain Monte Carlo draw. Posterior medians and 95% credible intervals were then derived for each municipality-month, together with the posterior probabilities $Pr\left(IRR_{it\_monthly} > 1\right)$. Maps display the posterior median IRR; when posterior samples were unavailable, maps display the IRR calculated from posterior mean fitted counts. As an internal consistency check, the population-weighted mean municipality-to-region IRR was verified to equal 1 within each month.

Results

Monthly spatial distribution of model-based risk. The municipality-to-region monthly IRR maps showed marked geographic heterogeneity in model-estimated human WNV incidence across Apulia. Values above 1 identified municipalities with fitted incidence higher than the corresponding regional monthly average, whereas values below 1 indicated lower fitted incidence. The spatial distribution and month-to-month evolution of posterior median IRR estimates are presented in **Supplementary Figure S1**. Posterior probabilities of an IRR greater than 1, indicating the strength of support for above-average municipal incidence, are reported in **Supplementary Figure S2**. These maps represent model-based relative incidence rather than observed transmission intensity. Elevated IRRs outside the observed transmission period should therefore not be interpreted as evidence of confirmed WNV circulation, but as model-derived spatial contrasts under the fitted spatiotemporal structure.

Figure S1. Monthly municipality-to-region IRR. Figure shows the monthly municipality-to-region model-based incidence rate ratio (IRR) for human WNV cases. Across the study period, model-estimated incidence was spatially heterogeneous, with several municipalities in northern Apulia and in the southern part of the region showing IRRs above the corresponding monthly regional average. The pattern was not restricted to the months in which human cases were observed; rather, moderately elevated IRRs were visible across much of the year, particularly in the northern cluster and in selected southern municipalities. During late summer and autumn, elevated IRRs remained evident in these areas, although most municipalities had IRRs below the regional monthly average.

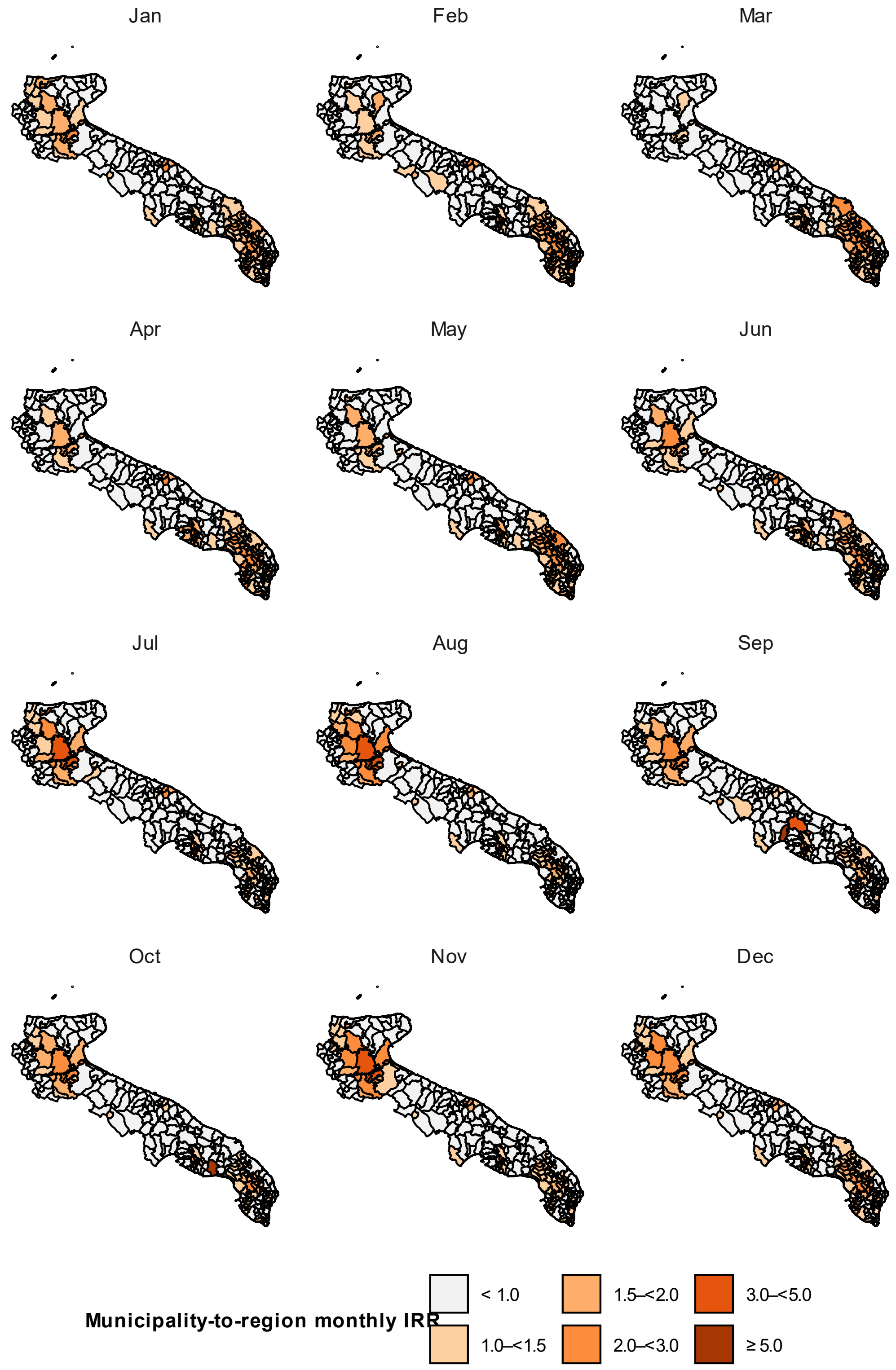

Figure S2. Posterior probability of elevated IRR. Figure shows the posterior probability that the municipality-to-region monthly IRR exceeded 1. Posterior support for above-average incidence was strongest and most spatially concentrated in southern municipalities during the early months of the year and again in late spring, whereas a northern cluster showed the most consistent high-probability pattern from July to November. In contrast, several municipalities with posterior median IRR above 1 had only intermediate posterior probabilities, indicating substantial uncertainty around the estimated elevation. Overall, the probability maps suggest that the apparent spatial pattern should be interpreted cautiously, particularly outside the areas with probabilities approaching 0.95 or higher.

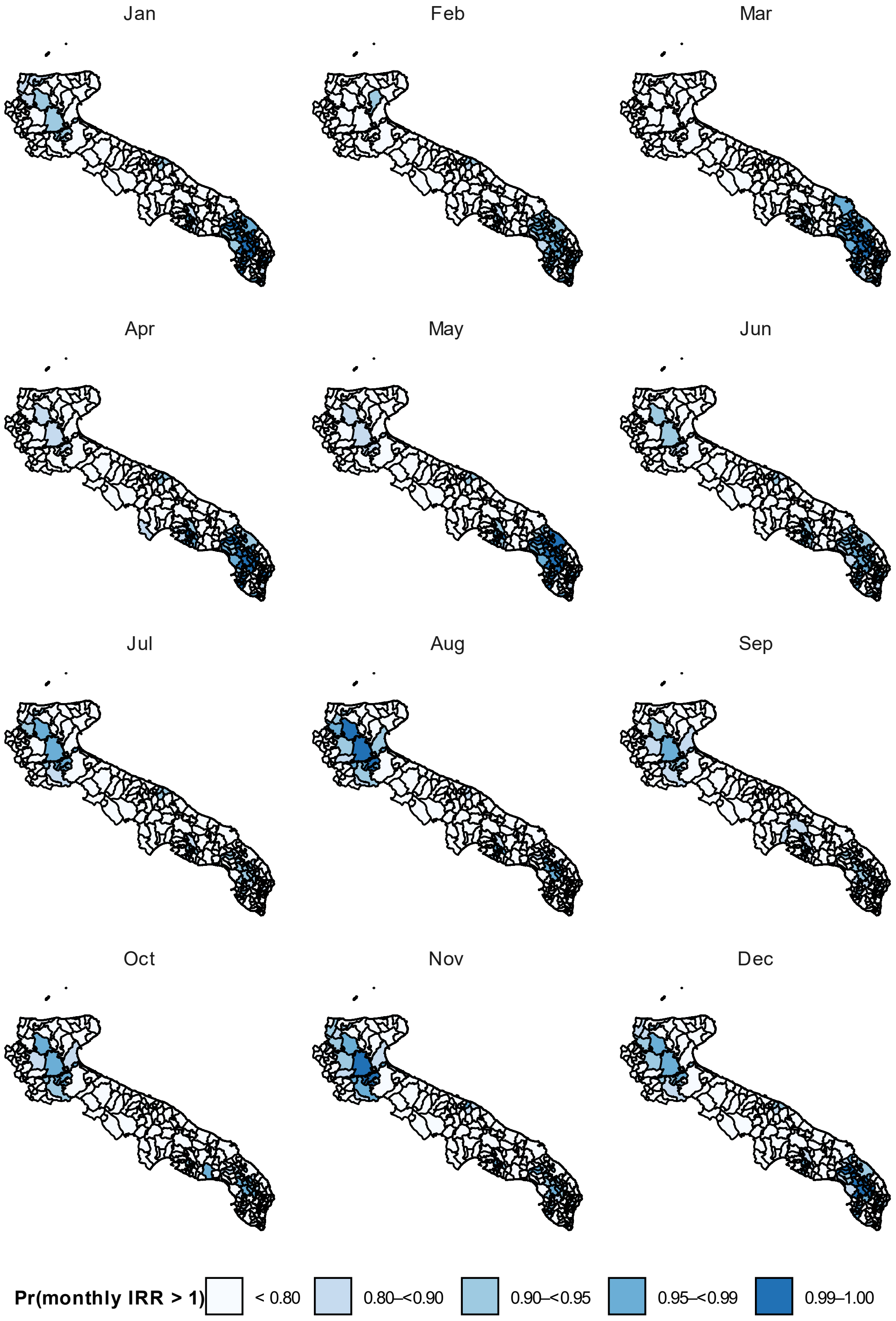

Supplement: Supplementary file 1 [file viruses-18-00943-s001.zip › viruses-4459157-supplementary.pdf]
